# Reconfigurable optoelectronic memristive architecture based on doped nanowire array for in-memory parallel perception and computation

**DOI:** 10.1093/nsr/nwaf386

**Published:** 2025-09-13

**Authors:** Lingchen Liu, Zhexin Li, Yiqiang Zheng, Linlin Li, Bowen Zhong, Yongchao Yu, Zheng Lou, Lili Wang

**Affiliations:** State Key Laboratory of Semiconductor Physics and Chip Technologies, Institute of Semiconductors, Chinese Academy of Sciences, Beijing 100083, China; Center of Materials Science and Optoelectronic Engineering, University of Chinese Academy of Sciences, Beijing 100049, China; State Key Laboratory of Semiconductor Physics and Chip Technologies, Institute of Semiconductors, Chinese Academy of Sciences, Beijing 100083, China; Center of Materials Science and Optoelectronic Engineering, University of Chinese Academy of Sciences, Beijing 100049, China; State Key Laboratory of Semiconductor Physics and Chip Technologies, Institute of Semiconductors, Chinese Academy of Sciences, Beijing 100083, China; State Key Laboratory of Semiconductor Physics and Chip Technologies, Institute of Semiconductors, Chinese Academy of Sciences, Beijing 100083, China; Center of Materials Science and Optoelectronic Engineering, University of Chinese Academy of Sciences, Beijing 100049, China; State Key Laboratory of Semiconductor Physics and Chip Technologies, Institute of Semiconductors, Chinese Academy of Sciences, Beijing 100083, China; Center of Materials Science and Optoelectronic Engineering, University of Chinese Academy of Sciences, Beijing 100049, China; State Key Laboratory of Semiconductor Physics and Chip Technologies, Institute of Semiconductors, Chinese Academy of Sciences, Beijing 100083, China; State Key Laboratory of Semiconductor Physics and Chip Technologies, Institute of Semiconductors, Chinese Academy of Sciences, Beijing 100083, China; Center of Materials Science and Optoelectronic Engineering, University of Chinese Academy of Sciences, Beijing 100049, China; State Key Laboratory of Semiconductor Physics and Chip Technologies, Institute of Semiconductors, Chinese Academy of Sciences, Beijing 100083, China; Center of Materials Science and Optoelectronic Engineering, University of Chinese Academy of Sciences, Beijing 100049, China

**Keywords:** reconfigurable architecture, persistent photoconductivity, low optical energy consumption, optoelectronic memristive, in-memory computation

## Abstract

Advanced hardware functional integration for emergent computing paradigms facilitates the potential optimization of computational redundancy in artificial intelligence. However, the device design for parallel perception and in-memory computing remains challenging. To develop a state-of-the-art integrated functional memory, this study demonstrates a reconfigurable optoelectronic memristive architecture (ROMA) based on a doped nanowire array for *in situ* parallel perception and in-sensor computation. The memristor based on In_2_S_3–X_As_X_ exhibits favorable photoconductive retention and reconfigurable optoelectronic modulation, which originates from vacancy engineering induced by doping modulation. The memristive performance of In_2_S_3–X_As_X_ can be tuned by controlling the doping dose. A monolithically integrated array demonstrates an improvement of the discriminative state by more than two orders of magnitude with a double sampling of the output signal, and achieves recognition and encoding of 12-bit binary optical signals on a single column. The nanowire memristive architecture provides an efficient hardware foundation for highly parallel and efficiently distributed computational paradigms.

## Teaser

A reconfigurable optoelectronic memristive architecture based on doped nanowire arrays for efficient, low-power in-memory computation and optical signal encoding.

## INTRODUCTION

The emergence of the Internet of Things and smart edge devices, coupled with the increasing prevalence of large artificial intelligence models, has led to substantial data processing demands. Current centralized computing paradigms based on the von Neumann architecture are inefficient for distributed, massively parallel and low-power computing models [1–3]. Although advanced machine learning algorithms have achieved significant efficiency improvement, inefficient hardware design without functional integration remains a major limitation [4]. To develop state-of-the-art hardware functional integration, memristive devices have attracted attention owing to their in-memory computing architectures with both storage and processing operations [5,6]. Memristive crossbar networks physically implement matrix–vector multiplication (MVM) with a high degree of parallelism, and their conductance could be reconfigurable by rules using modulation. However, the physical separation of perceptrons and memristors results in transmission bottlenecks, along with efficiency limitations and computational redundancy. Thus, the strategy of parallel perception and processing in memristor architectures requires further development.

As a type of super-speed perception medium, optical waves play a significant role in high-bandwidth and low latency transmission, and are expected to provide potential utilization in functional processing integration. The integration of optical perception in memristor architectures can overcome the transmission loss between sensors and memristor units, thereby accelerating machine learning and neuromorphic computing [7]. Current research on parallel optical perception in memristor architectures mainly focuses on bionic neuromorphic vision sensors [8,9]; however, the naturally unsustainable ability of retention and difficult weight modulation limit their application in optoelectronic computing. Optoelectronic memristors generally adopt the gate electrode to achieve persistent photoconductivity (PPC) and conductance modulation [10–13]. The presence of the gate electrode increases the complexity of integration. Therefore, it is necessary to develop an optimized optoelectronic memristive architecture with excellent retention and reconfigurable modulation to achieve highly integrated parallel functionality.

In this study, we propose a reconfigurable optoelectronic memristive architecture (ROMA) based on an In_2_S_3–X_As_X_-doped nanowire array for *in situ* parallel perception and information compression encoding in memory, where the ROMA had ultra-long photoconductivity retention characteristics of 6.819 × 10^5^ s. The optical power consumption of the excited optoelectronic memristive effect was less than 9.24 fJ. In_2_S_3–X_As_X_ possessed tunable characteristics and was able to bring about the transformation from volatile to non–volatile by regulating the doping amount. The ROMA demonstrates unique temporally resolved neuromorphic oscillations, and its conductivity weight could be modulated by a synergistic optoelectronic input. Reconfigurability in this context is a hierarchical concept, encompassing both device-level weight modulation and system-level functional adaptation. The mechanism underlying the reconfigurable memory in the device originates from the electron capture barrier induced by lattice relaxation, which is associated with doping-induced reductions in polar bond energy and changes in vacancy valence states. A monolithic integrated reconfigurable free-space optoelectronic encoder was fabricated to demonstrate optoelectronic computing in an In_2_S_3–X_As_X_ crossbar array. A 12-bit optoelectronic encoder was realized using a single nanowire in a single column with a coding accuracy of 92.8%. The device design of the ROMA and its monolithic integration provided an efficient reconfigurable hardware architecture for a distributed, highly parallel, low-power computational paradigm, which is expected to offer potential applications in optoelectronic computing.

## RESULTS

### Intrinsic characteristics and function of ROMA

**Figure 1.**
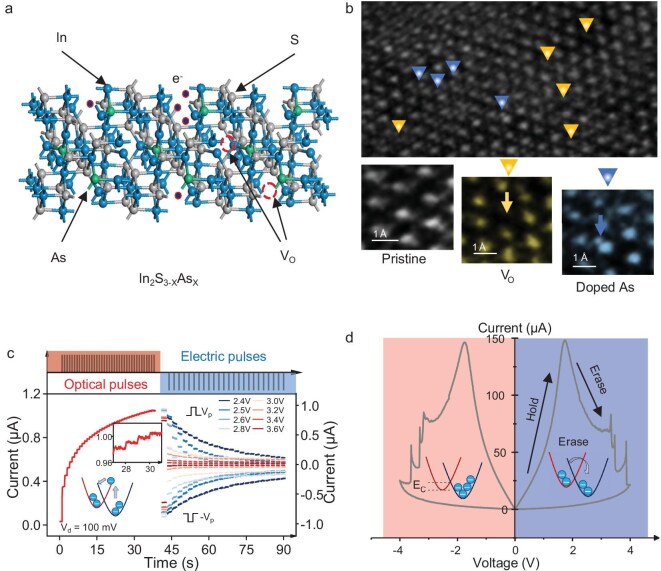
Reconfigurable optoelectronic memristive architecture based on In_2_S_3–X_As_X_ doped nanowire. (a) Schematic diagram of the crystal structure of In_2_S_3–X_As_X_ as the key functional material in this study. (b) HRTEM image of In_2_S_3–X_As_X_, inset showing surface oxygen vacancies and doped As atoms. (c) Characteristic photocurrent response of the device with optical pulse (30.84 μW cm^−^^2^, 100 ms) writing (left region) and voltage pulses (100 ms) for electrical programming at different amplitudes (right region). The first point on the right represents the current value after electrical pulse modulation. (d) Completely symmetrical *I*–*V* curve of optoelectronic memristor.

The ROMA demonstrates reconfigurable optoelectronic memristive characteristics based on the In_2_S_3–X_As_X_ nanowire, as shown in Fig. 1. The intrinsic crystallographic structure of the In_2_S_3–X_As_X_ nanowire is shown in Fig. 1a. The primary constituent of the material is In_2_S_3_ with minor incorporation of InAs, which is achieved through chemical vapor deposition (CVD), with a natural oxide layer on the surface [14,15]. It is confirmed using energy-dispersive X–ray spectroscopy (EDS) mapping (Fig. S1a and b). The PPC of In_2_S_3–X_As_X_ primarily arises from oxygen vacancies within the material’s surface oxide layer. In_2_S_3_ nanowires intrinsically develop an oxygen-rich surface layer (Fig. S1c), and InAs doping significantly amplifies the concentration of oxygen vacancies within this layer. The phenomenon visually corroborated by high-resolution transmission electron microscopy (HRTEM) imaging (Fig. S1d), where oxygen vacancies are indicated by yellow arrows and arsenic (As) dopant sites are indicated by blue arrows in the enlarged inset (Fig. 1b). This result shows a well-validated elemental composition. These resultant oxygen vacancies function as deep-level defect centers (DX centers), underpinning the material’s PPC and reconfigurable memristive characteristics.

Optical and electrical measurements are performed on the two-terminal device Au/Cr/In_2_S_3–X_As_X_/Cr/Au. Successive optical pulses are applied to the ROMA, and distinct non-volatile programmable multilevel conductance states are observed (Fig. 1c, left region), which can store more than 30 states (Fig. S2a). The enlarged view on the left in Fig. 1c and the tests of longer storage time under changing conditions (Fig. S3) verify the discrimination and stability of the multi-state storage of In_2_S_3–X_As_X_. The configuration diagram on the left of Fig. 1c demonstrates that after being illuminated by the optical pulse, the photogenerated electrons shift to the conduction band. Remarkable optical memory properties arise from the obstruction of the energy barrier when the photogenerated carriers at the conduction band edge (CBE) return to the ground state [16], which is significantly affected by As doping. The photoconductive state caused by optical excitation can be eliminated using voltage pulses (Fig. 1c, right region), and the conductance of the device is modulated by the number and amplitude of voltage pulses (100 ms). Additional details are illustrated in Fig. S2b, which shows the variation of the device conductance level with the number of voltage pulses and voltage amplitude (2.5–4.0 V). Unlike other memristors [17], the conductance modulation of the ROMA is related to the voltage amplitude and is independent of the voltage polarity. Therefore, it is completely symmetric when measuring the current–voltage (*I–V*) characteristic curve. This exhibits *I–V* characteristics with a giant hysteresis loop (Fig. 1d). This symmetric behavior is attributed to a field-enhanced carrier trapping mechanism. The electric field’s amplitude, regardless of its direction, provides the necessary energy to assist electrons in overcoming the capture barrier for recapture by deep-level DX centers. A 450 nm laser having a light intensity of 224 μW  cm^−^^2^ is used for irradiation before the *I–V* test, which indicates obvious optoelectronic memristor switching characteristics. The conductance of the device remains essentially unaltered at voltages below the switching threshold. The *I*–*V* curve shows a notable phenomenon of negative differential conductance above the switching threshold, and several negative differential conductance (NDC) regions appear as the voltage increases. The multiple NDC regions observed in Fig. 1d are attributed to a complex defect environment within the In_2_S_3–X_As_X_ nanowire, which presents the different height of carrier trapping barriers. As doping introduces a variety of deep-level defect states (DX centers) associated with lattice distortion and oxygen vacancies. This behavior is consistent with the Large Lattice Relaxation (LLR) model, where multiple trapping pathways with varying activation energies govern the carrier recombination dynamics. Notably, after the negative differential conductance, there is a slight curve of current that changes synchronously with voltage, the slope of which exhibits a decreasing trend, demonstrating that with increasing voltage, the conductance level of the device decreases continually until it returns to its initial state.

### Characterization of the optical and electrical properties of In_2_S_3–X_As_X_

**Figure 2.**
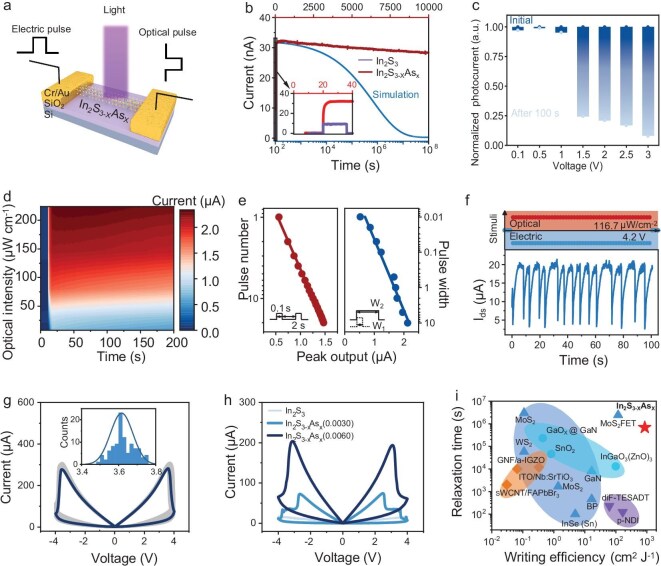
Excellent optical signal perception, PPC and programmability of light and electrical pulses based on In_2_S_3–X_As_X_. (a) Schematic diagram of an optoelectronic memristor based on In_2_S_3–X_As_X_. (b) Photocurrent curves for In_2_S_3–X_As_X_ and In_2_S_3_; the red curve represents the photocurrent relaxation curve of In_2_S_3–X_As_X_ (450 nm, 278 μW cm^−^^2^ for 5 s), the purple curve is for In_2_S_3_ (450 nm, 278 μW cm^−^^2^ for 15 s), the time coordinates of the two correspond to the red coordinate axis above, and the blue curve is the fitting result of In_2_S_3–X_As_X_ (V_d_ = 1 mV), which corresponds to the blue logarithmic coordinate axis below. (c) Normalized photocurrent diagram of the In_2_S_3–X_As_X_; dark and light blue dots represent the normalized photocurrent before and after voltage application, respectively. (d) The PPC curves (V_d_ = 100 mV) under different intensities of optical pulses (450 nm, 5 s). (e) Programmable properties of device conductance by light pulse (30.8 μW cm^−^^2^) number (left) and light pulse (30.8 μW cm^−^^2^) width (right). (f) *I*–*T* curve under constant light intensity and voltage (450 nm, 116.7 μW cm^−^^2^, V_d_ = 4.2 V). (g) The *I*–*V* cycling test curve graph, with positive and negative voltage cycling 100 times each; the inset shows the distribution of transition voltages. (h) The comparison diagram of *I*–*V* curves with different InAs doping contents (450 nm, 278 μW cm^−^^2^). (i) The comparison of the characteristic decay time and optical writing energy efficiency ratio of this study with previous research results.

Tests are conducted using a series of combinations of optical and electrical pulses to verify the optical and electrical modulation characteristics of the device. A schematic diagram of the device used in this study is depicted in Fig. 2a; it is a typical two-terminal device, which is different from the transistor structure that requires a gate to achieve PPC and conductance modulation. By eliminating the physical gate electrode and its associated control lines, this design reduces the device footprint and circuit complexity, enabling much higher integration densities to be essential for crossbar arrays. Furthermore, the two-terminal structure is naturally compatible with neuromorphic computing architectures, where it can seamlessly function as a weight in crossbar arrays to perform highly parallel in-memory MVM operations. Light is an important input source in the operational logic of the device, and there is a certain similarity to the gate in the physical sense, which can be directly used as an information input or for weight adjustment. Compared to the gate of a transistor, the optical input has a faster dynamic speed, lower latency, lower transmission loss, more free space and reconfigurable interconnects [7]. In this study, we mainly discuss light as the input information source for demonstration. The input voltage is as multifunctional as light, and the input optical information can be processed (e.g. calculated and encoded) using the voltage. Although it is a two-terminal device, it can achieve complex and diverse functions through the modulation of light and electricity, making it an attractive option for optoelectronic memristors. Notably, the perception and storage of optical signals by the In_2_S_3–X_As_X_ is in real time and the entire process of storage does not require an external voltage.

Long-term storage of optical signals is one of the main indicators of excellent optoelectronic memristors. The giant PPC phenomenon is observed in In_2_S_3–X_As_X_ after exposure to a 450 nm laser for 5 s at an intensity of 278 μW cm^−2^ (Fig. 2b); its photoconductance is more than two orders of magnitude higher than that of the dark state. The conductance of In_2_S_3–X_As_X_ decreases very slowly after the laser turns off, whereas that of In_2_S_3_ immediately returns to the dark state. By fitting the current relaxation curve with the Kohlrausch stretched exponential function, which is well suited for describing relaxation dynamics, we obtain a coefficient of determination (R²) of 99.51% [18]:


(1) \begin{eqnarray*}
I = ({I}_0 - {I}_\infty ) \times {\mathrm{exp}}[ - {(t/\tau )}^\beta ] + {I}_\infty,
\end{eqnarray*}

where I_0_ is defined as the illumination termination instant, I_∞_ is the value of the dark current, τ is the characteristic decay time constant and β is a decay index with a value between 0 and 1. The τ value of the fitting curve is approximately 6.819 × 10^5^ s, demonstrating excellent optoelectronic memristive properties, which is higher than the τ value of all reported two-terminal optoelectric memristors known to the authors.

Figure 2c illustrates the effect of different voltage amplitudes on the photoconductive properties of the device, which begins under the same initial conditions. The dark blue dots represent the normalized photocurrent at the moment when light was removed, and the light blue dot represents the normalized current after 100 s of light removal. The photoconductance level of the device remains relatively stable when the voltage is ≤1 V, which indicates that the photoconductance of the device has good retention characteristics over a wide range of voltages. The conductivity of the device significantly decreases when the external voltage is ≥1.5 V (Fig. S4a). This increasing field effect, which accelerates the PPC decay by increasing the electron-trap capture rate, is further evidenced by the voltage-induced hysteresis observed in the *I–V* characteristics (Fig. S4b). Figure S4c shows the *I*–*V* curve of the device in the initial dark state. Notably, as the voltage amplitude increases, the conductance amplitude of the device further decreases, exhibiting different, regular and distinguishable conductance levels. Figure S5 combined with Fig. S2b shows that the photoconductance of the device can be modulated by the amplitude of the applied voltage, number of voltage pulses, and width of the voltage pulses. Flexible and changeable modulation of device conductors can be achieved by the proper combination of different voltage variables. The modulation effect of the external applied voltage on the conductance of In_2_S_3–X_As_X_ is regarded as being that the electric field enhances the trapping probability of electron traps [19]. In addition, the device based on In_2_S_3–X_As_X_ exhibits excellent stability, repeatability and durability (Figs S6 and S7b).

The photocurrent curves of the device under different optical power densities irradiated by a 450 nm laser are shown in Fig. 2d, indicating that In_2_S_3–X_As_X_ has good response and retention properties for different optical power densities. The light response curves for additional wavelengths and intensities are shown in Fig. S8. Meanwhile, the *I*–*V* curves of In_2_S_3–X_As_X_ under different wavelengths are presented in Fig. S9. The UV–vis absorption spectrum of the In_2_S_3–X_As_X_ nanowires is presented in Fig. S10. Notably, the ability of the device to memorize optical signals becomes stronger with an increment in the light intensity (Fig. S11). When the light intensity is relatively low, the photoconductivity relaxation time of In_2_S_3–X_As_X_ will be significantly shortened (Fig. S12), manifesting the feature of a transition from non-volatile to volatile. In addition to the light intensity, both the number of optical pulses (Fig. 2e, left) and the optical pulse width (Fig. 2e, right) are programmed for device conductance (Fig. S13), which is similar to the modulation of voltage to conductance, and the programmed conductance has a stable retention performance. The fitting results show that the change in the photoconductance of the device is linearly related to the logarithm of the number of optical pulses and width of the optical pulses.

Memristor devices naturally exhibit high-order dynamics through internal electrophysical processes. The complex behavior realized by a single device can replace the function of hundreds or thousands of transistors. The interaction between devices can yield higher levels of functions and energy efficiency, and its native complex dynamics enable new computing architectures [20]. Currently, research in bionic computing is primarily based on simple memristor functions with first-order complexity, such as short-term plasticity, long-term plasticity, spatiotemporal convergence and summation. Second-order complexity components are uncommon [21,22], and there have been no reports on achieving second-order complexity in a single photoelectric memristor. The periodic oscillatory behavior of In_2_S_3–X_As_X_ in Fig. 2f is manifested under a specific set of conditions: continuous optical illumination (450 nm, 116.7 μW cm⁻²) combined with a constant, high DC voltage bias (4.2 V). The underlying mechanism is a dynamic equilibrium involving the periodic trapping and field-assisted de-trapping of electrons at deep-level Dx centers introduced by As doping.

In memristors, cycle durability and repeatability are essential indicators for assessing the performance of memristors. Distinct from general memristors that merely utilize electrical pulses for conductance modulation, the optoelectronic memristors synergetically regulate conductance using both optical pulses and electrical pulses. Thus, before the cyclic voltage test, irradiation with optical pulses is indispensable. We initially irradiate In_2_S_3–X_As_X_ with a 450 nm laser (278 μW cm^−^^2^), and then carry out 100 cycles of positive voltage and negative voltage tests, respectively (Fig. 2g), which manifests extremely good cycle stability and repeatability. In the inset of Fig. 2g, the distribution of transition voltages during 100 positive voltage cycles is presented, revealing excellent consistency. In addition to the high single-device endurance demonstration, device-to-device uniformity is also investigated. The results show promising consistency across different devices, including uniform photoresponse and consistent memory retention (Fig. S14).

In Fig. 2h, the comparison of the voltage cycling characteristics of In_2_S_3–X_As_X_ with different InAs doping amounts (0, 3, 6 mg) is presented. Before the test, it is irradiated with a 450 nm laser with a power of 278 μW cm^−^^2^. Through time-domain tests under a constant voltage, the effect of different InAs doping for the photoconductive retention performance of In_2_S_3–X_As_X_ is studied; the results demonstrate its tunable characteristic that can switch between volatile and non-volatile (Fig. S15). Experimental results demonstrate that increasing InAs doping significantly enhances device conductivity and memristive performance. Concurrently, the memristive switching voltage increases, which is likely due to the modulation of the electron capture barrier by InAs doping. Notably, InAs doping levels should be maintained within an optimal range; while excessive doping improves memristive performance of the device, the switching voltages increased by doping may compromise device endurance. Figure 2i summarizes the characteristic decay times and optical write efficiencies of In_2_S_3–X_As_X_ and previously reported 1D, 2D and bulk devices [18,23–35]. The optical write efficiency is the reciprocal of the light power density multiplied by the illumination time. Remarkably, among all devices, In_2_S_3–X_As_X_ exhibits the highest relaxation time and write efficiency, suggesting that In_2_S_3–X_As_X_ is more competitive for optical computing and optical memristors. We also analyzed the total energy consumption per operation, including both optical writing and electrical reading (Table S1).

### Mechanism analysis of In_2_S_3–X_As_X_

**Figure 3.**
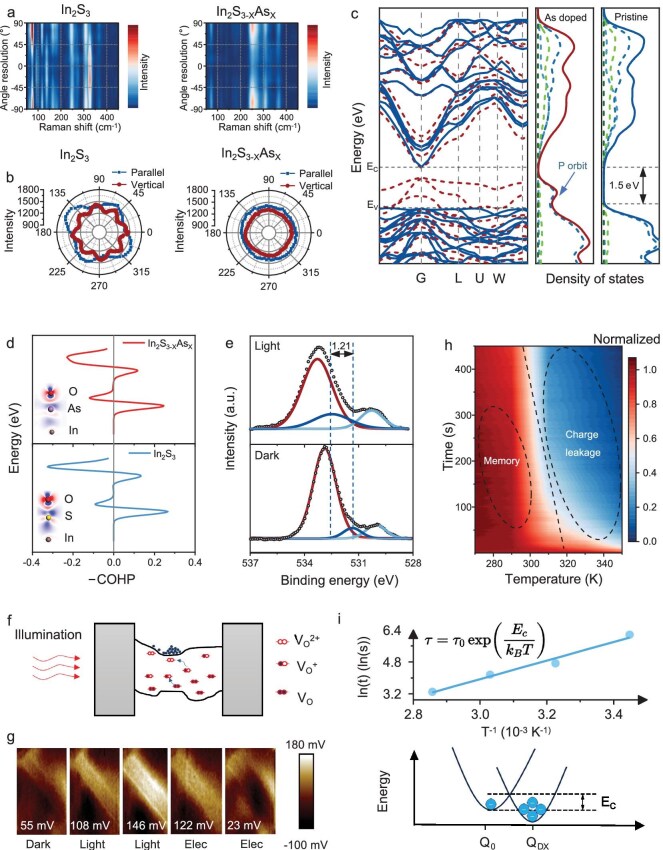
Characterization and theoretical calculation of In_2_S_3–X_As_X_. (a) Raman spectral mappings of In_2_S_3_ (left) and In_2_S_3–X_As_X_ (right) under different analyzer polarizations, showing the effect of As doping on vibrational modes. (b) Angle-resolved Raman scattering intensities of the Eg(2) mode for In_2_S_3_ (left) and In_2_S_3–X_As_X_ (right) in parallel and perpendicular configurations, indicating lattice symmetry changes due to doping. (c) Band structure and density of states curves of In_2_S_3_ (blue) and In_2_S_3–X_As_X_ (red) calculated using the DMol^3^ method. (d) COHP calculation results of indium–oxygen bonds in In_2_S_3_ and In_2_S_3–X_As_X_. (e) *In situ* XPS spectra of O1s of In_2_S_3–X_As_X_ in darkness and light. (f) Schematic diagram of the In_2_S_3–X_As_X_ under illumination; illumination empties the donor-like states while ionizing the oxygen vacancy sites, thus converting them from deep neutral states into shallow doubly ionized donor states. (g) Surface potential mapping of In_2_S_3–X_As_X_ nanowires measured by KPFM under sequential conditions: initial dark state, low-intensity light pulse, high-intensity light pulse, low-amplitude electrical pulse and high-amplitude electrical pulse. (h) Transient normalized PPC curves at different temperatures. (i) The resultant Arrhenius plot of the time constant for In_2_S_3–X_As_X_ (upper area), The CCD below shows the electron-capture barrier fitted by Eq. (2).

In_2_S_3_ and In_2_S_3–X_As_X_ exhibit large differences in their photoelectric responses (Fig. S16) and transistor performance (Fig. S17); the cause of these differences is of great interest. The Raman spectra (Fig. 3a) of In_2_S_3_ and In_2_S_3–X_As_X_ show significantly different peak positions and peak intensity changes when the analyzer polarization direction is changed (Fig. S18). The peak intensities of 93.8 and 326.2 cm^−^^1^ in In_2_S_3–X_As_X_ are significantly decreased compared to the Raman peaks of In_2_S_3_ (Fig. S19). The reduced intensity and broadened full width at half maximum (FWHM) of all the Raman modes are apparently due to the effect of As doping. In_2_S_3_ and In_2_S_3–X_As_X_ also show large differences in the parallel and perpendicular angle-resolved Raman spectra (Fig. 3b), indicating that As doping destroys the lattice symmetry of In_2_S_3_ [36]. As shown in Fig. S19, we found a significant blue shift of vibration frequencies in A_1g_ mode (248.2 cm^−^^1^), which can be ascribed to crystal lattice distortion by As doping and the substitution of surface sulfur vacancies by oxygen to form In–O when using CVD for As doping [14]. In_2_S_3_ and In_2_S_3-X_As_X_ were characterized using scanning electron microscopy (SEM) and X–ray diffraction (XRD), as shown in Fig. S20.

To further study the impact of As incorporation on the electronic structure of In_2_S_3_, first-principles calculations based on density functional theory (DFT) are performed. The band structure and density of states (DOS) of In_2_S_3_ and In_2_S_3–X_As_X_ are calculated using DMol^3^ (Fig. 3c and Note 1). The introduction of As has a significant impact on the p orbital of DOS in the valence band, and impurity energy levels are introduced in the valence band. The PPC phenomenon in In_2_S_3–X_As_X_ can be explained by the LLR model, which dominates the PPC phenomenon in crystalline semiconductors (Table S2). In the LLR model, the PPC effect is caused by an energy barrier that prevents the recombination of photogenerated electron–hole pairs (Note 2). This energy barrier is presumably caused by impurity atoms or donor vacancy complexes (usually called DX centers) [37]. When In_2_S_3–X_As_X_ is illuminated, the DX center is transformed into a shallow donor state with metastability, resulting in the creation of an energy barrier owing to the difference in lattice relaxation between the two states. The presence of DX centers in the semiconductor layer is responsible for PPC. In this case, the carriers are excited from the impurity centers with photon energies smaller than the bandgap [38].

According to previous studies, PPC is frequently related to vacancies in materials [16,39]. To verify that the oxygen vacancies caused by As doping have an influence on PPC, surface models of In_2_S_3_ and In_2_S_3–X_As_X_ are constructed. DMol^3^ is used to calculate the crystal orbital populations, and the crystal orbital Hamilton population (COHP) of the indium and oxygen atoms is obtained (Fig. 3d) [40]. The COHP diagram depicts the contributions of bonding and antibonding states to the energy of the band structure. The integral of the COHP (ICOHP) is calculated by integrating COHP within a given energy window. Values below the ICOHP Fermi level can be interpreted as the number of bonding electrons between atomic pairs, which can reflect the strength of the bond to a certain extent. In_2_S_3–X_As_X_ has a considerably lower indium and oxygen atom ICOHP than In_2_S_3_. This result demonstrates that oxygen atoms are more likely to separate from indium atoms to form oxygen vacancies because of As doping. When exposed to light with an energy close to or greater than the bandgap, one electron from the neutral oxygen vacancy is excited to the conduction band, leaving an ionized vacancy V_O_^+^ behind, thus liberating electrons that contribute to the PPC [41].

PPC in In_2_S_3–X_As_X_ is observed in the absence of oxygen, which excludes the mechanisms of oxidative chemical adsorption and photodesorption. PPC is more likely to originate from the photoionization barrier of oxygen vacancies in In_2_S_3–X_As_X_. Under illumination, neutral oxygen vacancies are ionized, producing electrons and ionized oxygen vacancies [V_O_^+^ or V_O_^2+^ (Eq. (2))]. When light is removed, an LLR occurs during the transition of V_O_^+^ and V_O_^2+^ to neutral oxygen vacancies, resulting in a potential barrier that prevents the recombination of photogenerated carriers. To further verify the role of the oxygen vacancies in In_2_S_3–X_As_X_ in PPC, In_2_S_3–X_As_X_ is characterized using *in situ* illumination X-ray photoelectron spectroscopy. In the O1s spectra (Fig. 3e and Fig. S21), there are abundant lattice oxygen (O_L_), oxygen vacancies (O_V_) and adsorbed oxygen (O_A_) [42]. Figure S7a shows that In_2_S_3–X_As_X_ has excellent PPC both in vacuum and in air (room temperature), indicating that the PPC of In_2_S_3–X_As_X_ is independent of oxygen in the air. The peak positions of O_V_ shift to higher binding energies after illumination. This phenomenon indicates that the oxygen in In_2_S_3–X_As_X_ produces photogenerated electrons under illumination, thereby producing ionized oxygen vacancies. V_O_^+^ and V_O_^2+^ are generally believed to be the origins of PPC characteristics; in particular, the PPC behavior results from the energy barrier required by the neutralization of ionized oxygen vacancies [43]. The PPC is primarily attributed to the ionization of oxygen vacancy (V_O_) sites; illumination with λ = 450 nm is thought to ionize the deep and neutral V_O_ states to shallow donor states (V_O_^2+^), as depicted in the band diagrams in Fig. 3f.


(2) \begin{eqnarray*}
\left\{ \begin{array}{@{}l@{}} V_{\mathrm{O}}^{\mathrm{0}} \to V_{\mathrm{O}}^{1 + } + 1e\\ V_{\mathrm{O}}^{1 + } \to V_{\mathrm{O}}^{2 + } + 1e \end{array} \right\}
\end{eqnarray*}

The synergistic optoelectronic control mechanism is directly visualized at the nanoscale using Kelvin probe force microscopy (KPFM) by sequentially imaging the nanowire’s surface potential under dark, light and electrical pulse conditions (Fig. 3g). During optical writing, the photo-ionization of surface oxygen vacancies [Eq. (2)] results in an accumulation of fixed positive charges (V_O_^2^^+^) on the nanowire surface. This charge accumulation induces upward band bending, which increases the work function of the surface and further raises the measured surface potential from a dark-state value of 55 mV to 146 mV [44]. Conversely, a subsequent electrical pulse initiates field-assisted carrier trapping, driving free electrons to neutralize these ionized vacancies. This neutralization of the fixed positive surface charge allows the upward band bending to relax, thereby decreasing the work function and reducing the measured potential to 23 mV.

Numerous studies have demonstrated that PPC is associated with an electron-capture barrier caused by the DX center [45,46], and its recovery is a thermally activated process. The photocurrent relaxation curves of In_2_S_3–X_As_X_ at different temperatures are measured using high- and low-temperature probe stations. The normalized photoconductance data for temperature dependence are shown in Fig. 3h and Fig. S22, and the Kohlrausch stretched exponential function in Eq. (1) can be used to extract the decay time constant *τ* at various temperatures [45]. Next, using the Arrhenius equation, the electron-capture barrier can be extracted by [47]:


(3) \begin{eqnarray*}
\tau = {\tau }_0\exp \left( {\frac{{{E}_c}}{{{k}_{\mathrm{B}}T}}} \right),
\end{eqnarray*}

where k_B_ is the Boltzmann constant and τ_0_ is the high T limit of τ. As shown in Fig. 3i, a linear least-squares fit to ln(τ) as a function of 1/T is employed to determine the electron-capture barrier E_c_, which is 404 meV.

The PPC effect in response to light and temperature is a hallmark of DX centers in semiconductors. The DX centers can switch to a charge-localized, electron-donating state by significant lattice relaxation when they are stimulated by light, and the electrons are excited to the conduction band. In the configuration coordinate diagram (CCD), lattice relaxation is usually described by displacement along the Q axis, reflecting the metastability of DX centers [48]. The electrons in the conduction band are blocked by the barrier E_c_ owing to lattice relaxation, resulting in the PPC effect. When the temperature increases, the electrons in the conduction band are thermally excited to cross the barrier E_c_ and return to the ground state; In_2_S_3–X_As_X_ also returns to the dark state conductivity.

### Free space photoelectric encoder based on ROMA

Memristors provide a higher level of integration, lower cost and better non-volatility than static random-access memory (SRAM) and dynamic random-access memory (DRAM) owing to their physical features. The memristor crossbar architecture, based on Ohm’s and Kirchhoff’s laws, can naturally execute MVM operations with a high degree of parallelism [49]. Coupled with the memory and weight tunable characteristics of the memristor, it has significant advantages for in-memory computing, artificial intelligence and neuromorphic computing. On this basis, an optoelectronic memristor can realize the perception of light and can be used for optical calculations; it allows for the advantages of low-loss transmission, free-space calculation, reconfigurable optical interconnects and one-way propagation.

**Figure 4.**
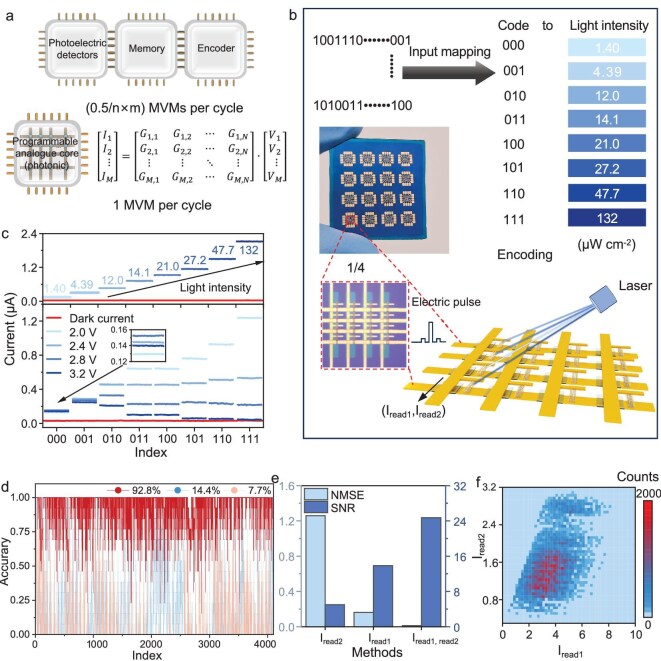
The free-space optoelectronic encoder based on ROMA. (a) Comparison between traditional photoelectric encoders and ROMA-based photoelectric encoders. (b) The working schematic diagram of the reconfigurable free-space optoelectronic encoder. (c) Optical and electrical modulation characteristics of ROMA; the upper part is the photocurrent value under eight light intensities (from 1.4 to 132 μW cm^−^^2^, 100 ms) encoded from ‘000’ to ‘111’, and the lower part is the corresponding current value after programming with voltage pulses (100 ms) with different amplitudes. (d) Decoding accuracy curve using three different output current data. (e) The comparison diagram of NMSE and SNR for encoding by solely using I_read1_, which denotes the sum of the output current under the original photoconductivity, encoding by exclusively employing I_read2_, which indicates the sum of the output current under the photoconductivity after electrical pulse modulation, and encoding through the combination of I_read1_ and I_read2_. (f) The distribution map of I_read1_ and I_read2_ in the 2D space, which contains 4096 groups of data (representing from 000000000000 to 111111111111), and each group encompasses 91 datapoints.

In traditional optoelectronic computing, an architecture with separate photodetectors, memories and processors is often adopted, which requires numerous sequential processing steps distributed across multiple cores for optoelectronic computing (Fig. 4a) [50,51], resulting in significant hardware costs, interface energy consumption and time delays [1]. An optoelectronic computing system based on In_2_S_3-X_As_X_ can realize the *in situ* perception, storage and calculation of optical information, and carry out highly parallel MVM operations, which greatly reduces the complexity of the circuit and the energy consumption of the interface.

Based on the tunable optical and electrical pulses of In_2_S_3–X_As_X_, we design a reconfigurable free-space optoelectronic encoder with an integrated crossbar array architecture. In this context, free space refers to the delivery of optical signals through an open medium (i.e. air) rather than via integrated physical conduits like waveguides. In Fig. 4b, the reconfigurable free-space photoelectric encoder array fabricated on SiO_2_/Si is depicted, each encompassing four 4 × 4 photoelectric encoders. Within the red dashed box lies a ^1^/_4_ magnified view of one of the devices, where each column constitutes a 12-bit photoelectric encoding unit composed of four In_2_S_3–X_As_X_ optoelectronic memristors. These memristors originate from the same nanowire and exhibit consistent performance (Fig. S14c). The schematic diagram on the right delineates the structure and working process of ROMA. Owing to the programmable feature of light intensity in In_2_S_3–X_As_X_, it can be categorized into eight disparate optical power densities in accordance with the photocurrent intensity response. The optical power densities, spanning from weak to strong, respectively correspond to ‘000’ to ‘111’. The operational workflow of the encoder begins by segmenting a 12-bit digital input into four parallel 3-bit packets, which are then converted into four optical pulses of corresponding intensities. These pulses are simultaneously projected onto a four-memristor column, where their intensities are stored as distinct conductance levels. A first readout (I_read1_) measures the initial summed current, primarily representing the total optical energy of the input signal. Subsequently, a vector of distinct programming voltages is applied to the corresponding rows, which reconfigurably modulates each memristor’s conductance. A second readout (I_read2_) captures the new summed current, which is now encoded with this sequence-dependent electrical weighting. Finally, compressed output is the 2D analog vector (I_read1_, I_read2_), which encapsulates both the intensity and sequence information of the original 12-bit signal.

The photocurrents corresponding to different optical power densities are presented in the upper part of Fig. 4c, and the reading voltage V_1_ was set at 0.1 V. In the reconfigurable free-space optoelectronic encoder, voltage pulses (2.0, 2.4, 2.8 and 3.2 V for 0.1 s) of varying amplitudes are applied to the memristor units in different rows. The modulation effect on photoconductivity is exerted by voltage pulses of different amplitudes (Fig. S23), which originates from the intrinsic properties within the material, can bestow different weights upon the memristor units and can be employed to distinguish the binary sequences in different rows. Furthermore, since the modulation effect of the same voltage pulse for different photoconductivity levels is also dissimilar (Fig. S24), this property in the material heightens the complexity of conductance modulation. After the application of the reading voltage V_2_ (V_2_ = 0.1 V), the current I_2_ of the memristor units following modulation by the voltage pulse is presented in the lower part of Fig. 4c. While the small photocurrent after modulation by the voltage pulse appears to overlap, the enlarged vision also distinguishes that there is a relative degree of distinction.

In ROMA, the sum of the output currents of the four memristor units in the same column under the original photoconductivity is denoted as I_read1_, and the sum of the output currents after being modulated by voltage pulses of different amplitudes is I_read2_. When encoding is conducted using only I_read1_, the average accuracy rate is merely 14.4% (Fig. 4d). When encoding is carried out using only I_read2_, the average accuracy rate is only 7.7%. To enhance the accuracy rate of encoding, on the one hand, I_read1_ needs to be used, and on the other hand, I_read2_ needs to be introduced, which contains the sequence information of four 3-bit binary sequences. The average accuracy rate when using both I_read1_ and I_read2_ as encoding information simultaneously reaches 92.8%. The substantial enhancement in encoding accuracy from single-readout values (14.4% for I_read1_, 7.7% for I_read2_) to 92.8% originates from the expansion of the encoding feature space from one to two dimensions. For using individually, I_read1_ primarily captures the total signal intensity and thus cannot distinguish sequences with similar energy, while the electrically modulated I_read2_ suffers from a compressed dynamic range that causes states to overlap. By using the vector (I_read1_, I_read2_) as the output, we leverage the synergistic optoelectronic properties of the device to create a unique 2D signature for each 12-bit input. This dual-signal approach resolves the ambiguities inherent in 1D measurements by simultaneously encoding both intensity and sequence information, dramatically improving the distinguishability between the 4096 possible states. This high performance is fundamentally enabled by the memristor’s high-resolution multistate storage capacity (Fig. S2a) and the complex non-linear dynamics governed by its defect-rich structure. This performance is directly linked to the synergistic optoelectronic modulation, multistate storage and complex internal dynamics of In_2_S_3–X_As_X_ memristors, making them highly suitable for optoelectronic computing tasks. Figure 4d presents the respective accuracy rates of 4096 states when the aforementioned three different encoding ways are utilized; the results verify that the ROMA can achieve the perception, storage and digital-to-analog conversion of 12-bit binary optical signals, and it can also realize the encoding and compression of information. At the same time, due to the internal complexity of the photoelectric modulation in the material itself, the encoding information is not easily cracked.

The normalized mean square error (NMSE) is a metric used to measure the similarity of image quality, defined as ||*v*-v*||2 2/||v*||2 2 for a vector, where *v* and v* represent the experimental and ideal vector, respectively. The signal-to-noise ratio (SNR) is defined as SNR = 10 × log_10_(I2 signal/I2 noise); it is a vital metric for assessing system performance and signal quality. Figure 4e and Fig. S25 show the NMSE and SNR when using I_read1_ alone, I_read2_ alone and the combination of I_read1_ and I_read2_ as encoding information. Among these, encoding with the combination of I_read1_ and I_read2_ had the lowest NMSE and the highest SNR. This is because the mapping interval of 4096 encoding results can be expanded from the 1D space to the 2D space, as shown in Fig. 4f, thereby increasing the information capacity of the unit interval (from N to N^2^). Thus, by making full use of the modulation properties of the material, a method has been proposed to improve the output data dimension, which can achieve the perception and storage of single 12-bit binary optical signals, significantly improving the accuracy of data encoding.

### The free-space optoelectronic encoder based on ROMA for the color image

**Figure 5.**
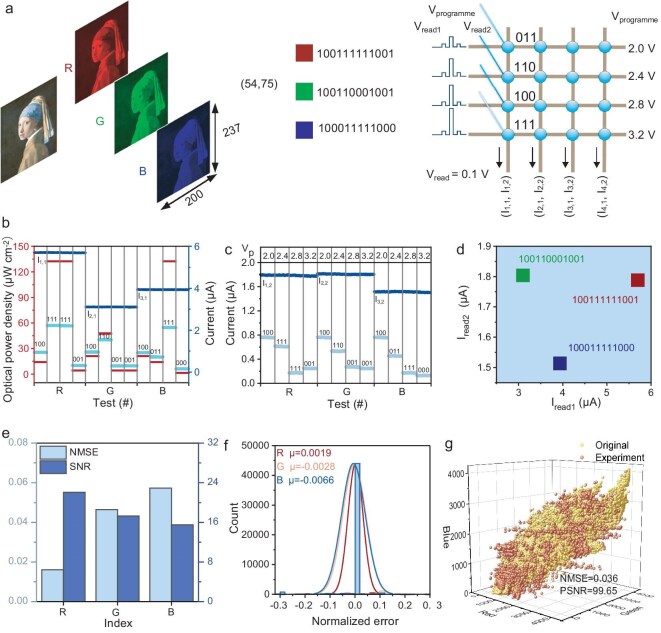
The free-space optoelectronic encoder based on ROMA for the color image. (a) Schematic diagram of photoelectric encoding for the color image (Mauritshuis, The Hague); the encoding process and performance are demonstrated by extracting the pixels with coordinates (54, 75) in the R, G and B space. (b) Schematic diagram of the optical power density, the photocurrent of a single device and the sum of the photocurrents of a single column corresponding to the R, G and B pixels with coordinates (54, 75). (c) The diagram of the current of each device and the sum of the currents of a single column after voltage pulse encoding. (d) The mapping diagram of the encoded output signals of the R, G, and B pixels with coordinates (54, 75) in the 2D space. (e) The schematic diagram of NMSE and SNR of the pixels in the R, G and B spaces of *Girl with a Pearl Earring* after passing through the photoelectric encoder. (f) The normalized error graph of pixels in the R, G and B space after encoding. (g) Comparison of original pixels and recovered pixels based on ROMA photoelectric encoder.

To visually demonstrate the encoding effect, we demonstrate the calculation process of converting a color picture (*Girl with a Pearl Earring* by Johannes Vermeer) into an optical signal and inputting it into an optoelectronic encoder (Fig. 5a). Color pictures can be divided into three color spaces, R, G and B; each color space is composed of 237 × 200 pixels, each pixel represented by a 12-bit binary number. We select the pixel point whose coordinate is (54, 75) in the R, G and B space as an example and represent it by 12-bit binary. Each 12-bit binary number is divided into four 3-bit binary combinations, and each set of the 3-bit binaries is encoded as a light pulse of the corresponding intensity during optical mapping. The encoded optical pulses are mapped to the optical encoder to realize the perception and storage of optical information. Three read–program–read voltage pulses (the read voltage pulses were 0.1 V, and the programmable voltage pulses were 2.0, 2.4, 2.8 and 3.2 V, respectively) are set up, and the sum of the currents in each column is obtained based on Ohm’s and Kirchhoff’s laws.

The first read voltage pulse resulted in an output of the sum of currents I_1,1_, I_2,1_ and I_3,1_ of the original photoconductance. Figure 5b shows the light intensities and photocurrents corresponding to the binary sequence of pixels in the R, G and B space during encoding, along with the sum of the photocurrents. Next, voltage pulses with varying amplitudes are employed to modulate the photoconductivity of the devices on different rows, enabling each device to have a distinct weight, so as to distinguish the sequence of the binary sequence.

The second read voltage pulse resulted in the output of the sum of currents I_1,2_, I_2,2_ and I_3,2_, after the photoconductivity is programmed, as shown in Fig. 5c. After being modulated by voltage pulses, the current amplitudes of I_1,2_ and I_2,2_ have a relatively small difference. However, due to the significant difference between I_1,1_ and I_2,1_ under the original photoconductivity, (I_1,1_, I_1,2_) and (I_2,1_, I_2,2_) have a significant degree of distinction. Under the original photoconductivity, I_3,1_ is significantly higher than I_2,1_, but after voltage pulse modulation, I_3,2_ is significantly smaller than I_2,2_. These results indicate the complexity of voltage pulse modulation. Using the free-space optoelectronic encoder, we realize the conversion of 12-bit binary digital information into two analog signals on each column. In Fig. 5d, we exhibit the distribution of the pixel points which are encoded in the R, G and B space and represented by 12-bit binary in the 2D space. Thus, via the ROMA-based photoelectric encoder, we map the information represented by the binary sequence onto the points in the 2D space, attaining the compression and storage of signals. Furthermore, the inherent complexity of the optoelectronic modulation imparts a high degree of hardware-intrinsic security to the information encoded by the ROMA.

By utilizing the ROMA-based free-space photoelectric encoder, we carry out encoding tests on the pixels in the R, G and B color space of the *Girl with a Pearl Earring* image, and quantify the encoding results of different color spaces by means of NMSE and SNR (Fig. 5e). The results indicate better encoding quality. Figure 5f shows the normalized histogram of the calculation errors obtained by subtracting the recovered pixels from the original pixels in the R, G and B color space of the *Girl with a Pearl Earring* image. The histogram is fitted using the Gaussian distribution, and the mean values of the normalized errors in the R, G and B space are all extremely low, verifying the accuracy and stability of the ROMA-based free-space photoelectric encoder. Figure 5g shows the distribution of recovered pixels and original pixels in 3D space. One indicator of the recovery quality is the peak SNR (PSNR), which is defined as 10 log_10_(4095^2^/mean square error (MSE)). Compared with the original pixels, the experimental recovery pixels encoded by the ROMA show an NMSE of 3.6 × 10^–2^, and the PSNR is 99.65 dB. The coding accuracy of the R, G and B color space of the picture is shown in Fig. S26. Notably, the free-space photoelectric encoder based on ROMA has extremely good encoding accuracy.

## CONCLUSION

In this study, we fabricated a ROMA and monolithic integration based on an In_2_S_3–X_As_X_ nanowire array for *in situ* parallel perception, storage and memory computing. The ROMA demonstrated a favorable retention of approximately 6.819 × 10^5^ s and programmable characteristics of synergistic optoelectronic modulation, which achieved complex multilevel modulation in the ROMA at both terminals. The intrinsic memory and reconfigurable modulation were attributed to the valence change of vacancies owing to the electron-capture barrier of the DX centers and the reduction of polar bond energy from As doping. Monolithic integration was fabricated to design a reconfigurable free-space optoelectronic encoder based on the memristor crossbar architecture, achieving a 12-bit optoelectronic encoder on a single row by improving the output data dimension, with a coding accuracy of 92.8%. The device design of the ROMA and its monolithic integration provide an efficient reconfigurable hardware architecture for distributed, highly parallel, low-power computational paradigms, and is expected to offer potential applications in optoelectronic computing.

## METHODS

### Material synthesis

In_2_S_3–X_As_X_ nanowires were synthesized on 300 nm SiO_2_/Si substrates using a two-step atmospheric pressure CVD method. Initially, a 3 nm Au film was deposited on the substrate via thermal evaporation to serve as a catalyst. During the first CVD step (BTF-1200C-III-S, Anhui Beiyike Equipment Technology Co., Ltd, China), an alumina boat containing In_2_S_3_ powder (Alfa, 99.999%) was positioned at the center of a quartz tube, while the Au-coated SiO_2_/Si substrate was placed downstream, approximately 16–18 cm from the precursor. Argon (Ar) was employed as the carrier gas, with the flow rate set to its maximum for 20 min to purge residual gases from the quartz tube. Subsequently, the flow rate was adjusted to 100 sccm. The furnace temperature was ramped from ambient to 900°C over 30 min, maintained for 2 h to facilitate nanowire growth, and then naturally cooled to room temperature. For the second CVD step, an alumina boat containing InAs powder (Alfa, 99.9999%) was placed at the center of the quartz tube, with the substrate bearing In_2_S_3_ nanowires positioned downstream, approximately 18–20 cm from the precursor. The carrier gas flow rate was maintained at 100 sccm, and the growth was conducted at 900°C for 1 h, followed by natural cooling.

### Device fabrication

A standard probe station with a semiconductor analyzer (B1500A, Keysight) was used to evaluate the optoelectronic properties of the fabricated nanowire devices, using a laser with adjustable output pulse width and power as the light source. Temperature-dependent electrical measurements were performed under high vacuum (∼1 × 10^−4^ Pa) using a variable temperature probe station (Lake Shore Cryotronics).

### Material characterization

TEM with an EDS analyzer (JEM-2010F) was used to characterize the lattice structure and composition of the In_2_S_3–X_As_X_ nanowires. The morphologies of the nanowires were observed by SEM (Nova NanoSEM 650). The XRD data were acquired using an X-ray powder diffractometer (Bruker D8 ADVANCE, λ = 1.5418 Å). A confocal Raman spectrometer with a laser excitation wavelength of 532 nm (WITEC alpha300R) was used to record Raman and PL spectra. The XPS measurement was carried out by using a Thermo ESCALAB 250Xi analysis system. The surface potential of In_2_S_3–X_As_X_ under different light intensities was characterized by KPFM (Bruker Dimension Icon scanning probe microscope). The UV–vis absorption spectrum was measured by Shimadzu UV-3600 with dual detector integrating sphere.

### Fabrication of ROMA

Horizontal memristor crossbar electrodes were deposited on a SiO_2_ substrate by thermal evaporation. Al_2_O_3_ was deposited on the substrate as a dielectric layer by atomic layer deposition, and excess parts were removed by wet etching. The vertical memristor crossbar Cr/Au electrodes were deposited on the substrate by thermal evaporation, and the In_2_S_3–X_As_X_ nanowires were transferred to the memristor crossbars, then the contact electrodes were deposited, and finally the excess nanowires were removed by dry etching.

### DFT simulation

DFT calculations are performed using DMol^3^ code. The structure and electronic properties of In_2_S_3–X_As_X_ and In_2_S_3_ were calculated using the DMol^3^ code. The electron exchange-correlation functional was described by the generalized gradient approximation (GGA) using the Perdew–Burke–Ernzerhof (PBE) functional. The DFT semi-core pseudopots (DSPP) and the double numerical basis sets with polarization (DNP) functions were adopted in all calculations.

## Supplementary Material

nwaf386_Supplemental_File
