# Supplementary material for: Reconfigurable optoelectronic memristive architecture based on doped nanowire array for in-memory parallel perception and computation
Source: Natl Sci Rev. 2025 Sep 13;12(11):nwaf386. doi: 10.1093/nsr/nwaf386 (PMC12598564; doi:10.1093/nsr/nwaf386)
Supplement: nwaf386_Supplemental_File [file nwaf386_supplemental_file.pdf]

Supplementary Data

Reconfigurable optoelectronic memristive architecture based on doped nanowire array for in–memory parallel perception and computation

Lingchen Liu[1,2], Zhexin Li,[1,2]*, Yiqiang Zheng[1], Linlin Li[1,2], Bowen Zhong[1,2], Yongchao Yu[1]*, Zheng Lou[1,2]*, Lili Wang[1,2]*

[1] State Key Laboratory of Semiconductor Physics and Chip Technologies, Institute of Semiconductors, Chinese Academy of Sciences, Beijing 100083, China

[2] Center of Materials Science and Optoelectronic Engineering, University of Chinese Academy of Sciences, Beijing 100049, China

* Corresponding authors

E-mails: lizhexin193@semi.ac.cn (Z. X. Li), yuyc126@semi.ac.cn (Y. C. Yu), zlou@semi.ac.cn (Z. L.) liliwang@semi.ac.cn (L. L. W.)

This PDF file includes:

Figs. S1 to S26

Note 1-3

Table S1-S2

Note 1. DFT simulation. In this study, $In_2S_{3-X}As_X$ demonstrates giant persistent photoconductivity and photoelectrically adjustable characteristics. This is attributed to the effect of arsenic doping on the properties of In_2S_3 nanowires. Therefore, calculating the changes in electronic properties caused by arsenic doping will help understand the mechanism of the phenomenon. The electronic structure calculations and geometry optimizations of In_2S_3 and $In_2S_{3-X}As_X$ are carried out using the Density Functional Theory (DFT) implemented in the Dmol3 code [1]. The generalized gradient approximation (GGA) is adopted to describe the exchange correlation interaction with the parameterization by Perdew, Burke, and Enzerhof (PBE) [2]. The DFT Semi–core Pseudopots (DSPP) and the double numerical basis sets with polarization functions (DNP) are adopted in all calculations. Grimme method is used for DFT–D correction. Unrestricted spin-polarized self–consistent field (SCF) calculations are carried out. The Brillouin zone is sampled with $5\times5\times5$ irreducible Monkhorst–Pack k–points grid for the structural relaxation and for the electronic density of states. SCF tolerance is set to 10^{-6} eV/atom. Geometry optimizations are performed with a convergence criterion of 1×10^{-5} Ha for total energies, 2×10^{-3} Ha/ Å for the maximum force and 5×10^{-3} Å for the maximum displacement.

Researches show that metal doping added to the β–In_2S_3 lattice, such as Mn [3], V [4], Nb [5], Ag [6] etc., can introduce impurity energy levels in the energy band gap. Through comparative analysis of the band structures and densities of states of In_2S_3 and $In_2S_{3-X}As_X$, the doping of arsenic element introduced impurity energy levels in

the valence band. Doping plays an important role in controlling and manipulating the electrical conductivity of materials by creating various energy levels. It can manifest as shallow traps and deep traps, with deep traps making a significant contribution to the PPC phenomenon [7].

Note 2 Mechanism elaboration of $In_2S_{3-X}As_X$. For There are currently many model explanations for the origin of the PPC phenomenon, mainly including the macroscopic barrier model (MB) [8], the large lattice relaxation model (LLR) [9] and the random local potential fluctuation model (RLPF) [10]. In general, LLR dominates in crystalline semiconductors, while RLPF and MB models dominate in disordered semiconductors [11]. Through the high-resolution electron transmission and electron diffraction photos of $In_2S_{3-X}As_X$ in Fig. S1d, as well as the XRD test result (Fig. S20b), it is proved that the $In_2S_{3-X}As_X$ nanowire is a single crystal.

In the LLR model, the PPC originates from deep–level traps, known as the DX center. When the light illuminates, the DX center is converted into a metastable shallow donor state such that a potential barrier is created due to the difference in lattice relaxation between the two states. The PPC effect is caused by a barrier that prevents electrons from being recaptured at the DX center. The schematic diagram of the LLR model is shown in the lower subplot in Figure 3i, where Q_{DX} and Q_0 represent the ground and excited states, respectively, and E_C is the height of the barrier. When the light illuminates, the carriers are excited from Q_{DX} to Q_0. When the light is removed, the carriers need to cross the barrier E_C from Q_0 back to Q_{DX}, which leads to the PPC phenomenon. The recovery of PPC is a thermally activated process. The

recovery of PPC is a hot-activated process. Therefore, through the testing of the high and low temperature probe station and the Arrhenius equation, the barrier E_C of the $In_2S_{3-X}As_X$ is calculated to be 404 meV.

There are many control methods for PPC, such as excitation light parameters, applied electric field, temperature, polarization, etc. The most important of these are the excitation light parameters–wavelength, pulse width and intensity. By controlling these parameters, PPC can be used for short–term memory (STM), long–term memory (LTM) and spike timing dependent plasticity (STDP) [12]. The applied electric field is also an important regulatory parameter, which is able to regulate the photoconductance of the device. When the electric field is strong, the PPC formation process will be hindered and the decay process will be accelerated. The electric field effect is thought to be caused by an increase in the capture rate of electron traps, which may be related to the deep central Coulomb repulsion of PPC [13].

Note 3 Operational workflow of the reconfigurable free–space optoelectronic encoder. The encoder is built upon a monolithically integrated crossbar array of $In_2S_{3-X}As_X$ memristors. A single column, comprising four memristor devices, have functions as a complete 12–bit encoding unit. These memristors, originating from the same nanowire, exhibit high performance consistency, which is crucial for reliable encoding.

Input Mapping: The encoding process begins with the digital–to–optical conversion of the input signal. A 12–bit binary digital input is first segmented into four parallel 3–bit packets. Each 3–bit packet, representing a value from "000" to

"111", is mapped to one of eight pre–defined optical power densities (ranging from 1.4 to 132 μW cm^{-2}). This creates four parallel optical pulses, with the intensity of each pulse encoding the value of its corresponding 3–bit packet.

Synergistic Optoelectronic Encoding Process: The core of the workflow involves a multi–stage process that utilizes both optical and electrical stimuli to encode the input signal.

Phase I: Parallel Optical Perception and Intensity Sampling (I_{read1}) The four optical pulses generated in the previous step are simultaneously projected onto the four memristors of the target column. Each device perceives its corresponding light pulse and, due to the material's persistent photoconductivity (PPC), its conductance is set to a unique level proportional to the incident light intensity. This achieves parallel perception and in-memory storage of the optical information. Immediately following this, a first readout is performed to capture the intensity information of the signal. A low read voltage (V_1 = 0.1 V) is applied across the column, and the total summed current, designated I_{read1}, is measured in accordance with Ohm's and Kirchhoff's laws.

Phase II: Reconfigurable Electrical Programming for Sequence Encoding. To encode the sequential order of the four 3–bit packets, a vector of distinct programming voltage pulses (e.g., 2.0 V, 2.4 V, 2.8 V, and 3.2 V for 100 ms) is applied to the four corresponding rows of the crossbar array. This action modulates the conductance of each of the four memristors by a different amount, effectively applying a unique electrical weight to the stored optical information.

The magnitude of this modulation depends on both the voltage pulse amplitude and the initial photoconductivity level of the device, making the process highly non-linear and sequence-dependent.

Phase III: Sequence-Encoded Readout (I_{read2}). After the electrical programming step, a second readout is performed. The same low read voltage (V_2 =0.1 V) is applied to the column again, and the new summed current, designated I_{read2}, is measured. This I_{read2} value now reflects the state of the memristors after the sequence-dependent electrical modulation.

Dual-Sampling and High–Accuracy Compressed Output: The final step of the workflow is the creation of the compressed output. The encoder adopts a dual-sampling strategy, where the two measured values, I_{read1} (intensity) and I_{read2} (sequence), are combined to form a single 2D analog vector (I_{read1}, I_{read2}).

This method expands the encoding capacity from a one–dimensional (N–state) domain to a two–dimensional (N^2state) domain, which significantly improves the distinguishability of the 4096 possible 12–bit binary combinations. As visualized in the 2D distribution map (Fig. 4f), this dual–signal approach results in well–separated data clusters, enabling a final encoding accuracy of 92.8%. This process successfully achieves high–precision information compression and encoding within a single, integrated hardware unit.

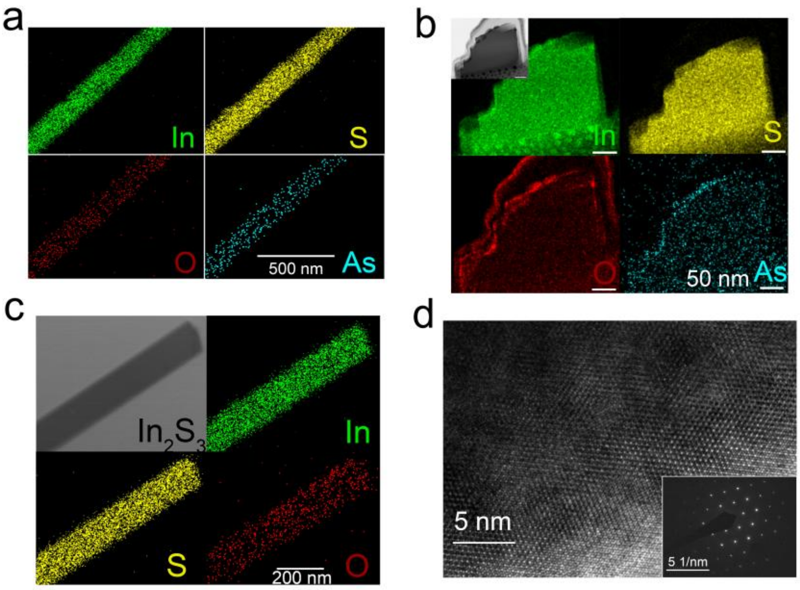

Fig. S1 Characterization of $In_2S_{3-X}As_X$ and In_2S_3 nanowires. (a) The EDS mapping of $In_2S_{3-X}As_X$ demonstrates the successful doping of arsenic into the In_2S_3 nanowires. (b) Cross–sectional EDS mapping of $In_2S_{3-X}As_X$ nanowires. (c) EDS mapping of individual elements in In_2S_3. (d) The HRTEM image of $In_2S_{3-X}As_X$, the illustration is electron diffraction pattern.

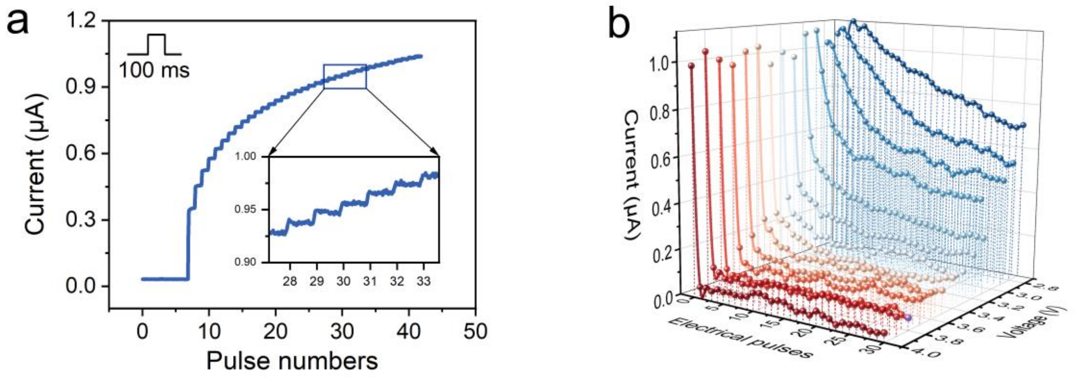

Fig. S2 Modulation effect of optical and electrical pulses on ROMA. (a) The optical pulses (30.84 μW cm^{-2}, 100 ms) program the conductance of the ROMA and are capable of achieving more than 30 different conductance states. (b) The programming effect of different amplitude and number of voltage pulses (100 ms) on ROMA conductance, it can be seen that voltage pulses of different amplitudes have different erasing conductance states.

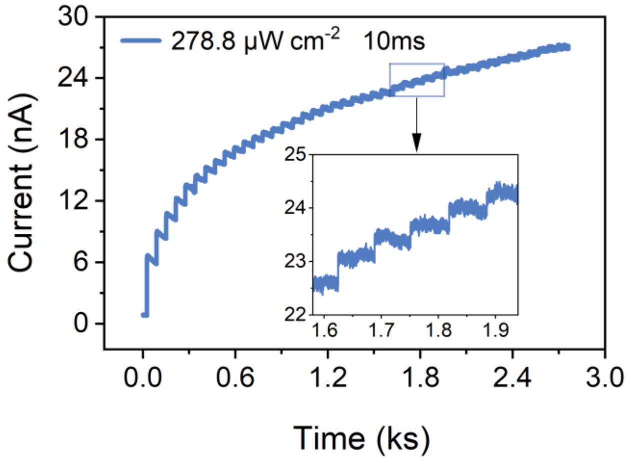

Fig. S3. The multi–states and stability of photo–pulse encoding. Under the light pulse of 450 nm (278.8 μW cm^{-2}, 10 ms), the $In_2S_{3-X}As_X$ exhibits excellent multi–state encoding capabilities, and each state has good discrimination and long-term stability.

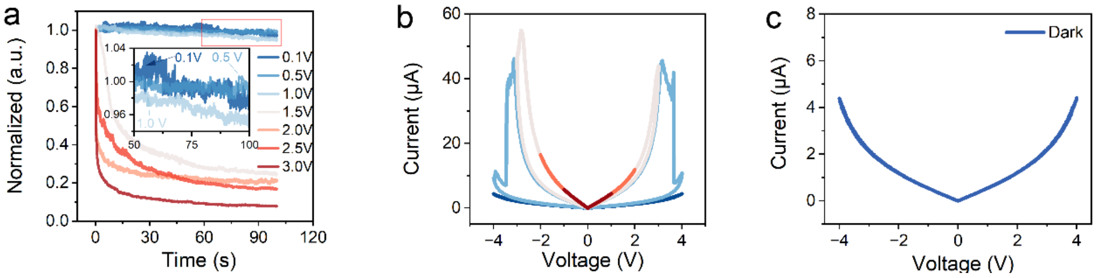

Fig. S4 Effect of voltage on $In_2S_{3-X}As_X$ photoconductivity. (a) Time dependence of $In_2S_{3-X}As_X$ normalized current values under different voltage amplitudes. (b) *I*–*V* curves of $In_2S_{3-X}As_X$ under different voltage amplitudes, as the scan voltage amplitude increases, a hysteresis window appears in the *I*–*V* curve, and the conductance level decreases. (c) The *I*–*V* curve of $In_2S_{3-X}As_X$ in the dark state.

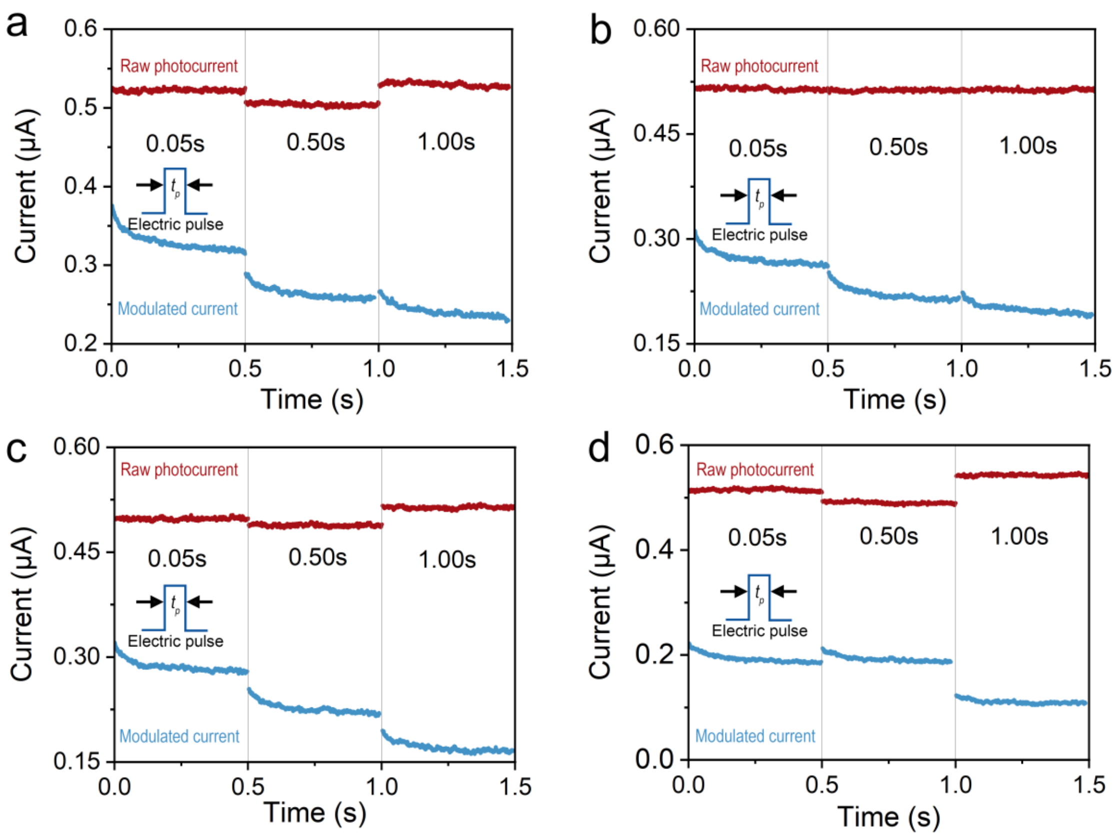

Fig. S5 Effect of electrical pulse width on photoconductivity. The programming effect of voltage pulses with different pulse widths on ROMA when the voltage amplitude is 3.0 V (a), 3.1 V (b), 3.2 V (c) and 3.3 V (d) respectively. The red curve represents the photocurrent of the device following photoexcitation, while the blue curve indicates the photocurrent after modulation by electrical pulses.

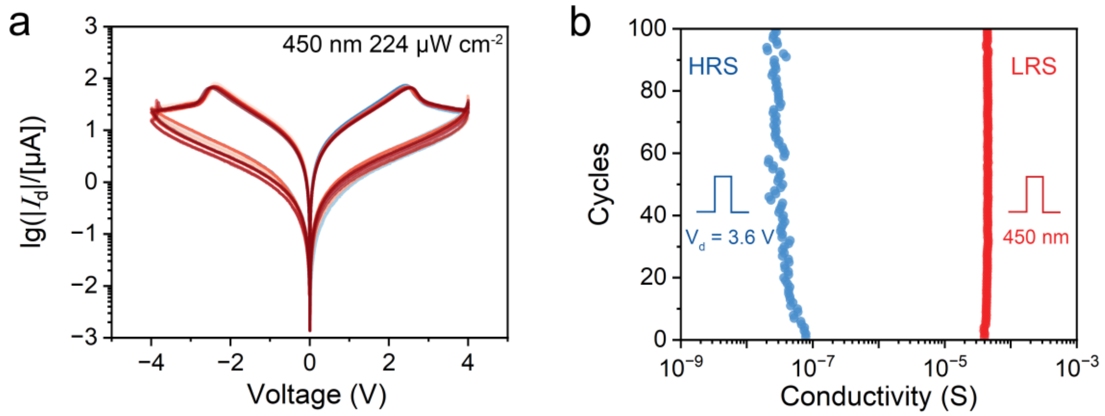

Fig. S6 Repeatability and durability of $In_2S_{3-X}As_X$. (a) A total of 46 *I*–*V* curves (including positive and negative voltage scans) were tested on $In_2S_{3-X}As_X$. Prior to each test, it was irradiated with a laser having an intensity of 224 μW cm^{-2}, and the outcomes exhibited excellent consistency. (b) Experiment involving the writing (indicated by red dots) with a light pulse (450 nm, 224 μW cm^{-2}) and the erasing (indicated by blue dots) with an electrical pulse (V_d = 3.6 V) of $In_2S_{3-X}As_X$.

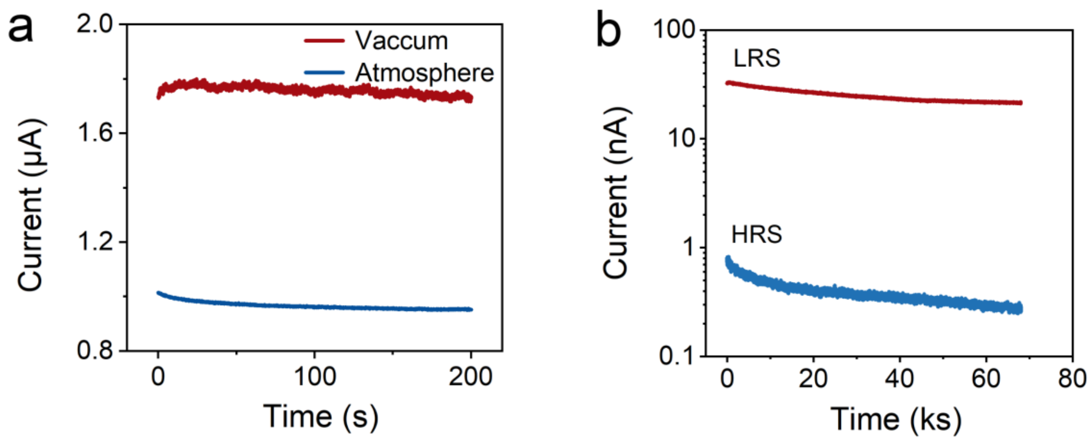

Fig. S7 The photoconductivity retention test of $In_2S_{3-X}As_X$. (a) The photoconductance retention characteristics of $In_2S_{3-X}As_X$ after exposure to 450 nm in vacuum and air were compared at room temperature (Vd = 100 mV). (b) A schematic diagram illustrates the stability of $In_2S_{3-X}As_X$ in the high-resistance state (HRS) and low-resistance state (LRS) at Vd = 1 mV. The HRS corresponds to the device state after reset by a voltage pulse (4.2 V, 1 s), while the LRS reflects the enhanced photoconductive state following excitation by a light pulse (450 nm, 224 μW cm^{-2}, 5 s).

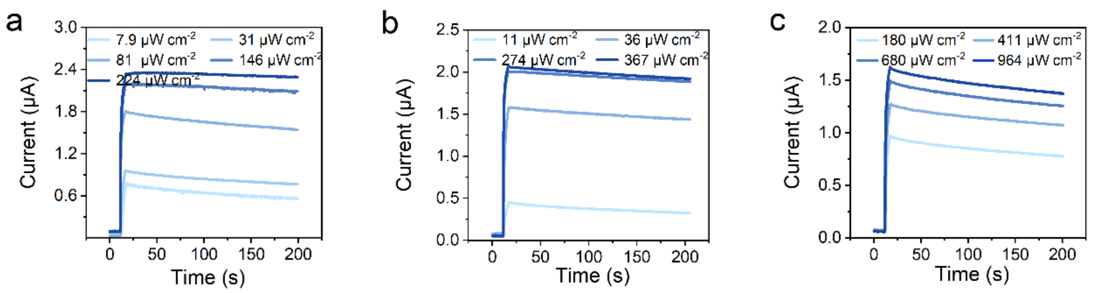

Fig. S8 Photoconductivity retention characteristic curves at different wavelengths. (a) (b) (c) Photoresponse and photoconductivity retention curves at 450 nm (a), 532 nm (b) and 635 nm (c) wavelengths

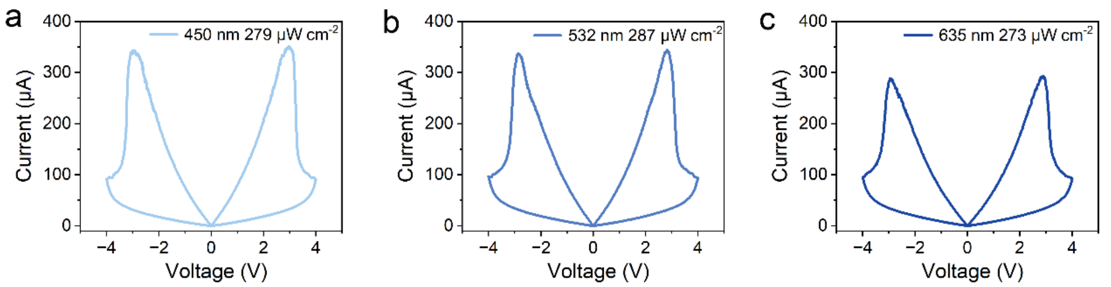

Fig. S9 *I*–*V* characteristic curves at different wavelengths. (a) (b) (c) The *I*–*V* characteristic curves at 450 nm (a), 532 nm (b), and 635 nm (c) wavelengths.

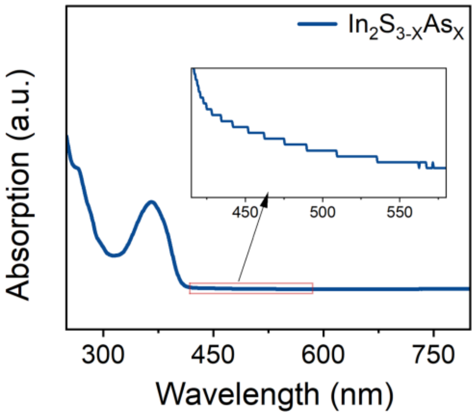

Fig. S10 UV–VIS absorption spectra of $In_2S_{3-X}As_X$, with the absorption band edge around 400–420 nm.

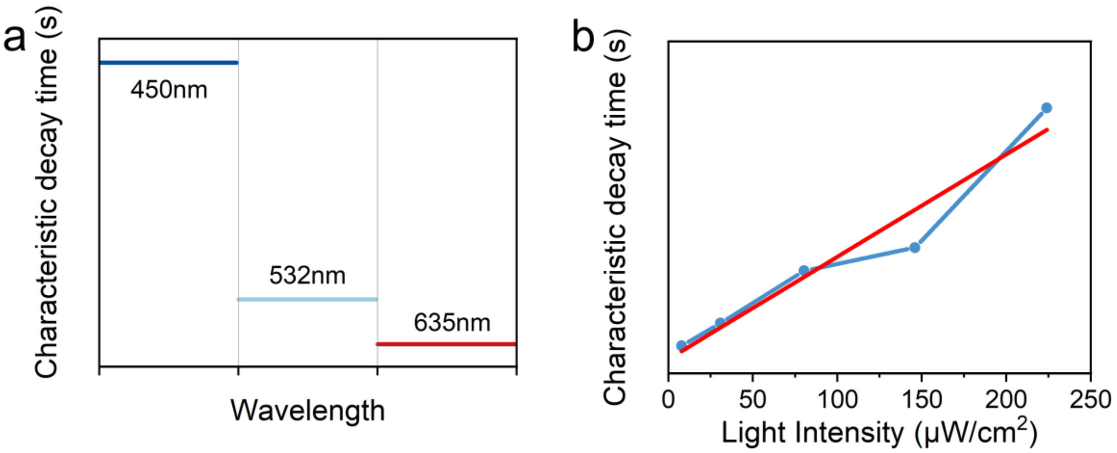

Fig. S11 Effects of wavelength and light intensity on the characteristic decay time constant of $In_2S_{3-X}As_X$. **a** Characteristic decay time (τ) of different wavelengths (Light intensity is almost the same). **b** Effect of different optical power densities on characteristic decay time (τ) at 450 nm wavelength.

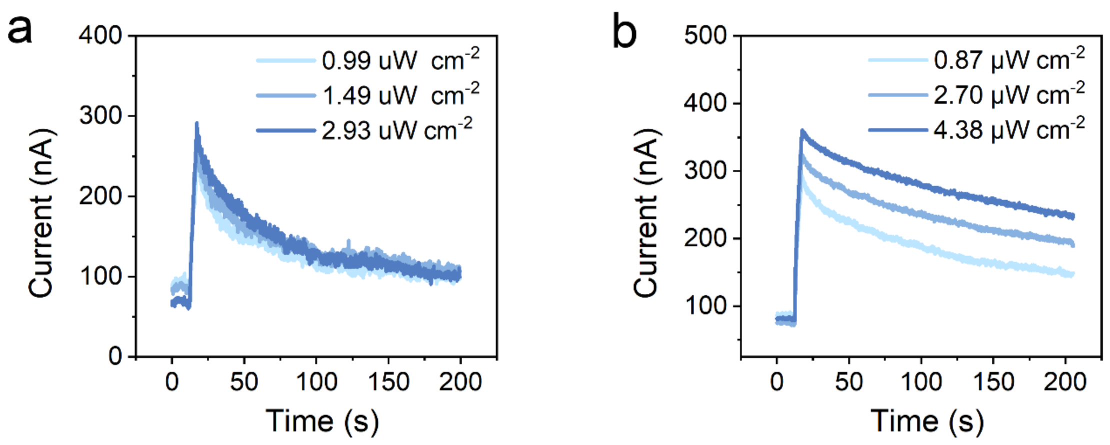

Fig. S12 Photoconductance retention characteristic curves of $In_2S_{3-X}As_X$ under low light intensity. (a) The photoconductivity relaxation curve of $In_2S_{3-X}As_X$ under a 450 nm light pulse (5 s) shows minimal change in photocurrent at lower light intensities. This could be attributed to the combined effects of insufficient carrier generation and the presence of trap states under these conditions [14–15]. (b) Under a 532 nm light pulse (5 s), the photoconductivity relaxation curve of $In_2S_{3-X}As_X$ exhibits a higher photocurrent compared to that at 450 nm, despite similar light intensities. This enhancement may be due to the superior absorption efficiency of $In_2S_{3-X}As_X$ at 532 nm, influenced by its bandgap and trap states [6,15-16].

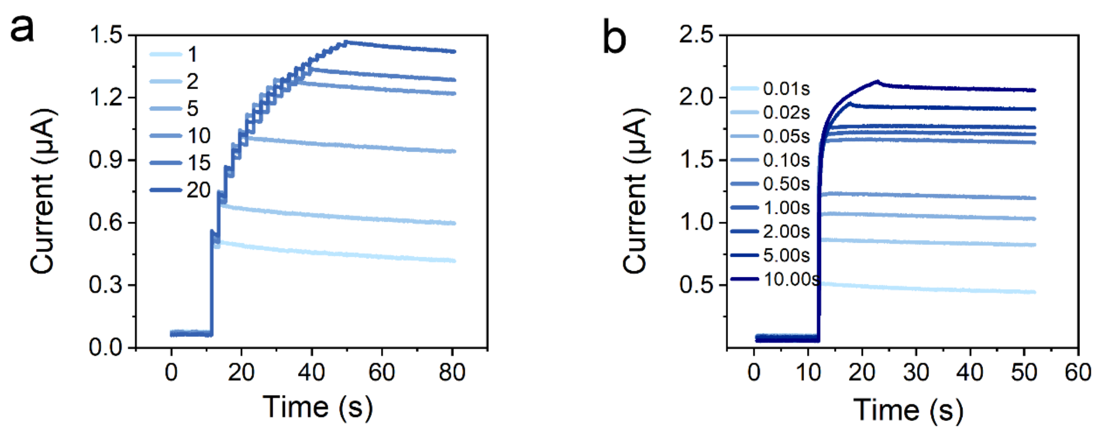

Fig. S13 Effect of light pulses on conductance. (a) Effect of the number of light pulses on the photoconductivity state of $In_2S_{3-X}As_X$. (b) Modulation of $In_2S_{3-X}As_X$ photoconductivity by optical pulse (30.8 μWcm^{-2}) width.

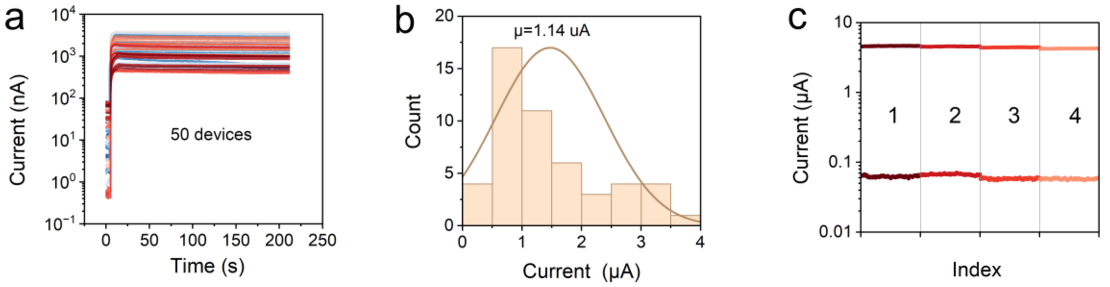

Fig. S14 Consistency of the $In_2S_{3-X}As_X$ sample. (a) Photoconductivity retention characteristic curves of 50 $In_2S_{3-X}As_X$ nanowire devices. (b) Statistical distribution of photocurrent responses of 50 devices under the same light pulse conditions. (c) Optical pulse writing and electrical pulse erasing curves at 4 different locations on the $In_2S_{3-X}As_X$ nanowire.

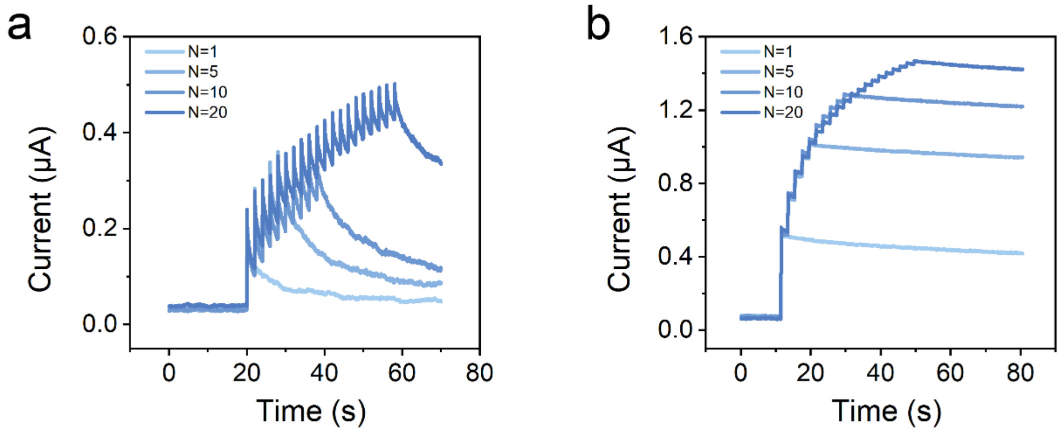

Fig. S15 Optical pulse modulation curves of $In_2S_{3-X}As_X$ with different doping contents. (a) **a** Optical pulse (450 nm at 30.84 μW cm^{-2} for 10 ms) modulation curve of $In_2S_{3-X}As_X$ at 3 mg doping amount of InAs. (b) Optical pulse (450 nm at 30.84 μW cm^{-2} for 10 ms) modulation curve of $In_2S_{3-X}As_X$ at 6 mg doping amount of InAs.

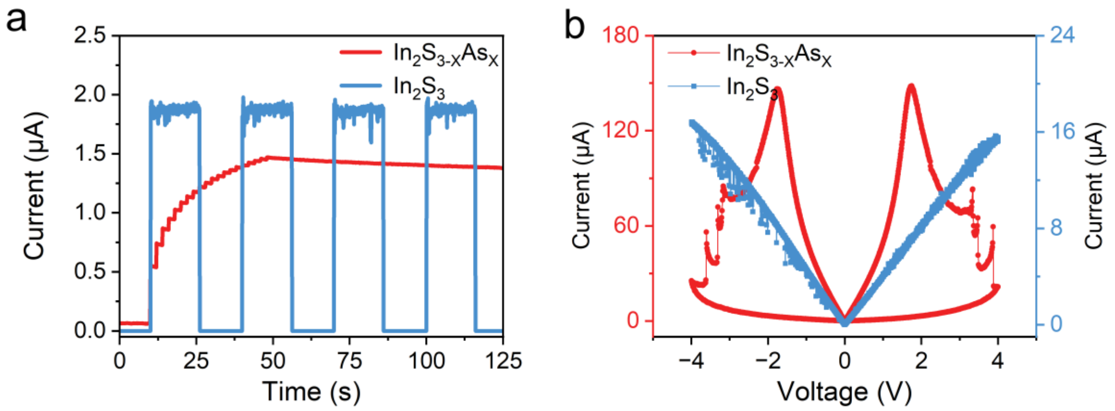

Fig. S16 Comparison of photoelectric properties of $In_2S_{3-X}As_X$ and In_2S_3. (a) *I–T* curves of $In_2S_{3-X}As_X$ and In_2S_3 under light pulses, $In_2S_{3-X}As_X$ has remarkable optical pulse modulation and photoconductivity retention properties. (b) *I–V* curves of $In_2S_{3-X}As_X$ and In_2S_3 under light, compared with In_2S_3, the *I-V* curve of $In_2S_{3-X}As_X$ exhibits obvious memristive switching characteristics.

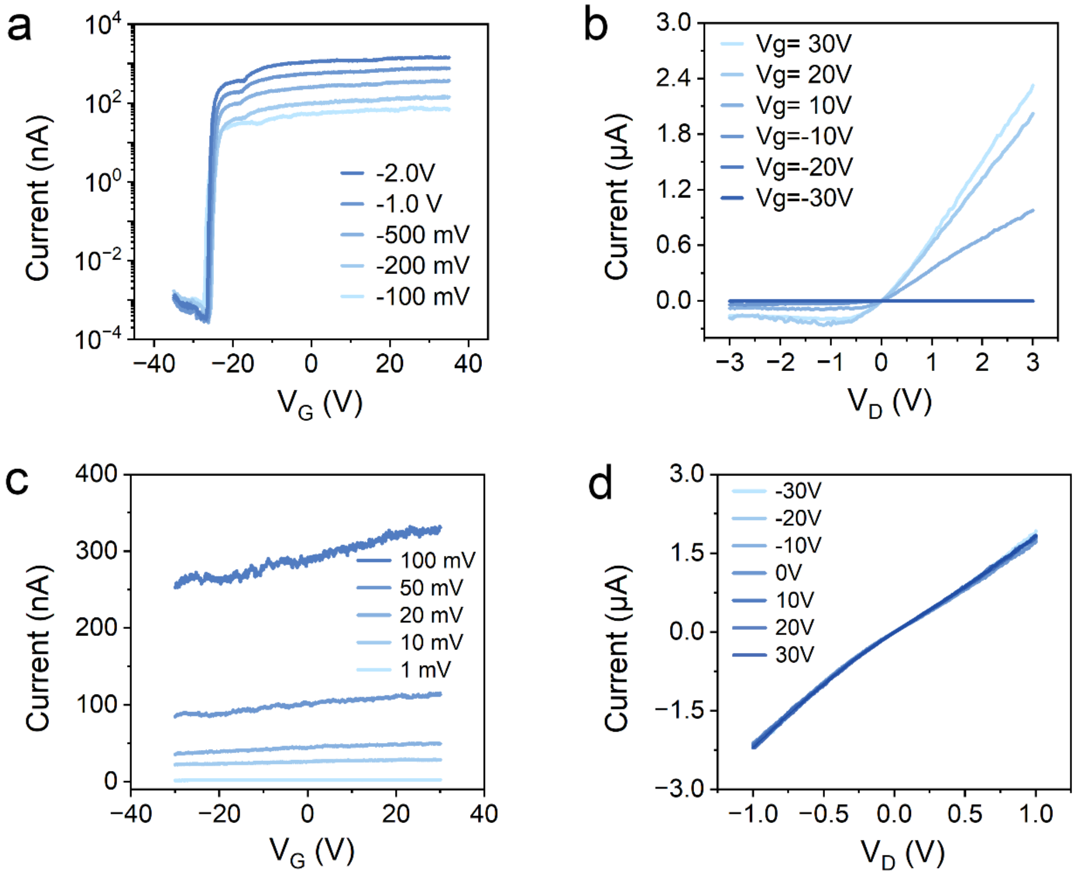

Fig. S17 Transistor performance comparison between In_2S_3 and $In_2S_{3-X}As_X$. (a) (b) Plots of the transfer and transmission characteristics of In_2S_3. (c) (d) Plots of the transfer and transmission characteristics of $In_2S_{3-X}As_X$.

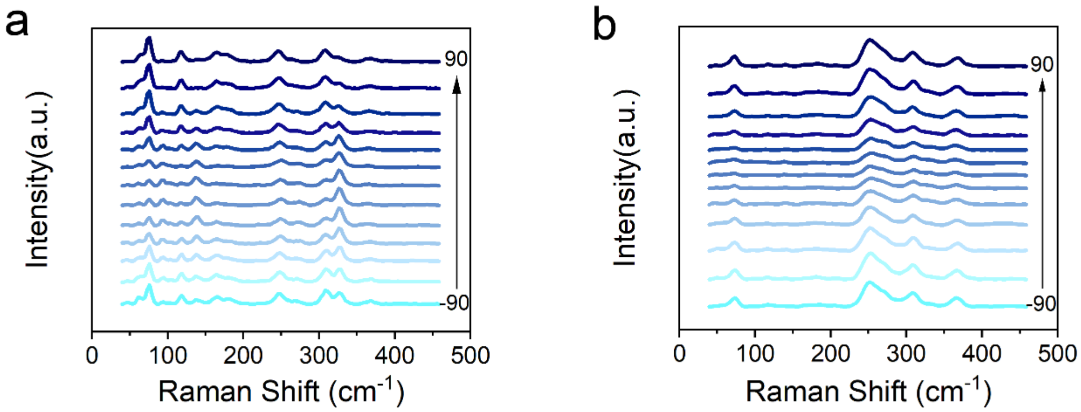

Fig. S18 Raman spectrum of In_2S_3 and $In_2S_{3-X}As_X$. (a) (b) When the laser polarization angle remains unchanged and the polarization angle of the analyzer is adjusted from –90 degrees to 90 degrees (detected every 15 degrees), the Raman spectra of In_2S_3 (a) and $In_2S_{3-X}As_X$ (b).

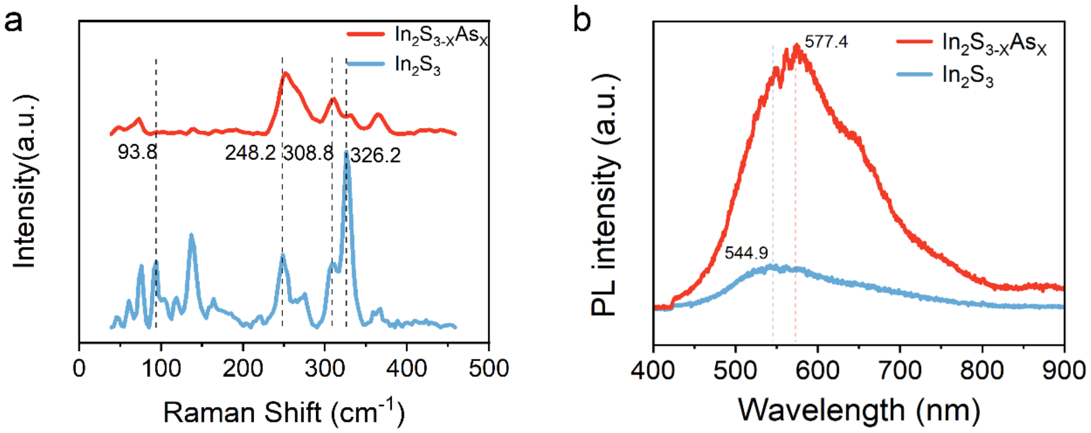

Fig. S19 Comparison of Raman and PL spectra of In_2S_3 and $In_2S_{3-X}As_X$. (a) Comparison of Raman spectra of In_2S_3 and $In_2S_{3-X}As_X$ in the perpendicular ($E_{\perp}$) direction. (b) Comparison of PL spectra of In_2S_3 and $In_2S_{3-X}As_X$.

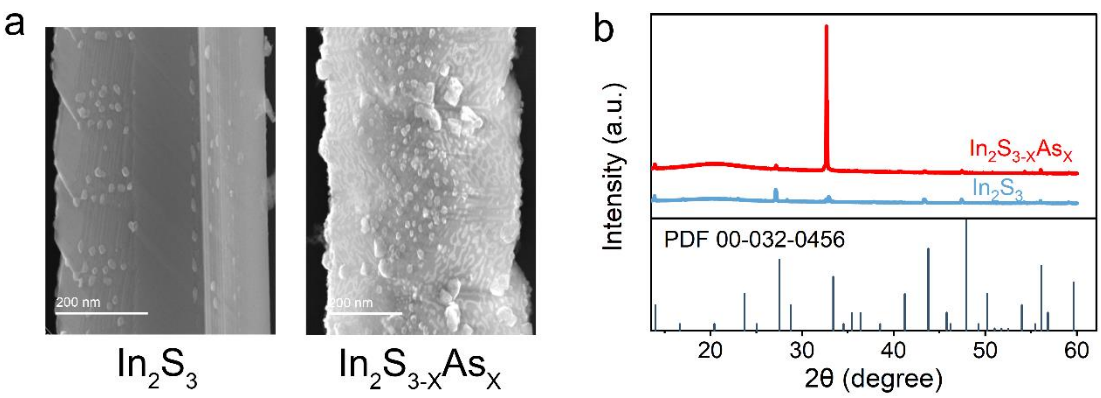

Fig. S20 Sample characterization of In_2S_3 and $In_2S_{3-X}As_X$. (a) Morphology of In_2S_3 and $In_2S_{3-X}As_X$ under scanning electron microscope (SEM). (b) The upper part is the X-ray diffraction patterns of In_2S_3 and $In_2S_{3-X}As_X$, and the lower part is the standard PDF card diagram of In_2S_3.

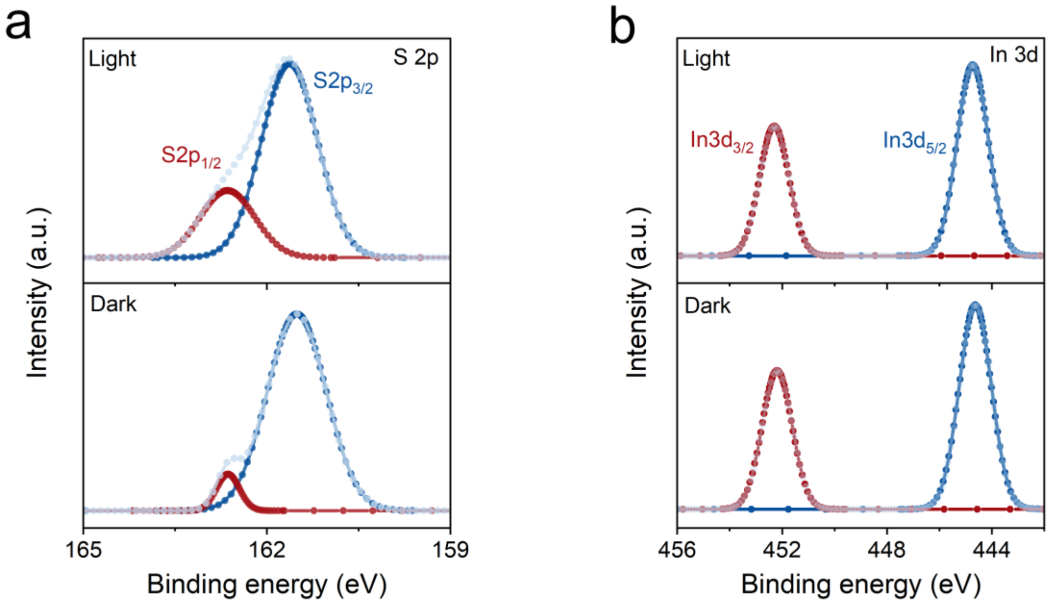

Fig. S21 XPS spectra of $In_2S_{3-X}As_X$ sample. XPS spectra of S 2p (a) and In 3d (b) for $In_2S_{3-X}As_X$.

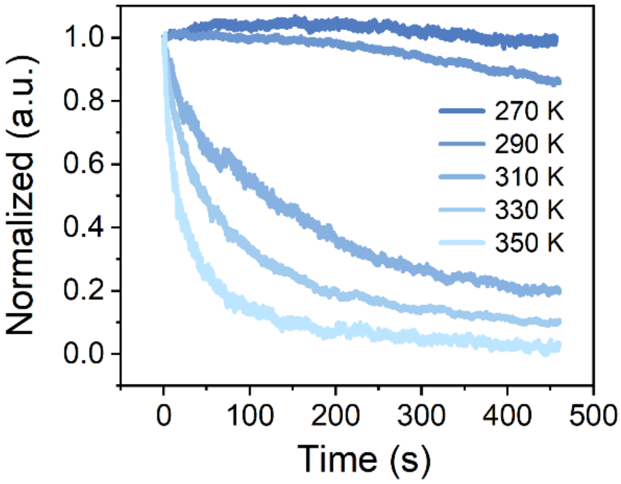

Fig. S22 Normalized PPC curves of $In_2S_{3-X}As_X$ at different temperatures.

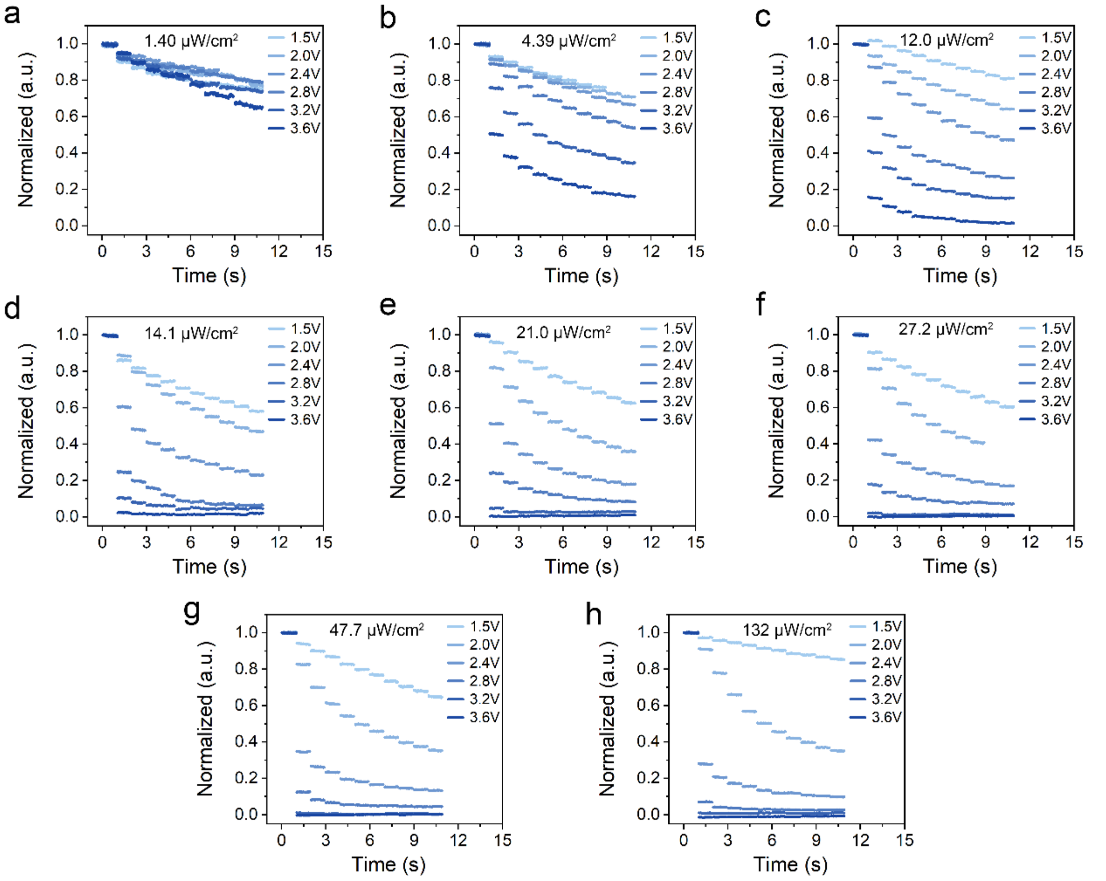

Fig. S23 Effect of voltage pulse (100 ms) on photoconductivity of $In_2S_{3-X}As_X$ excited by different optical power densities.

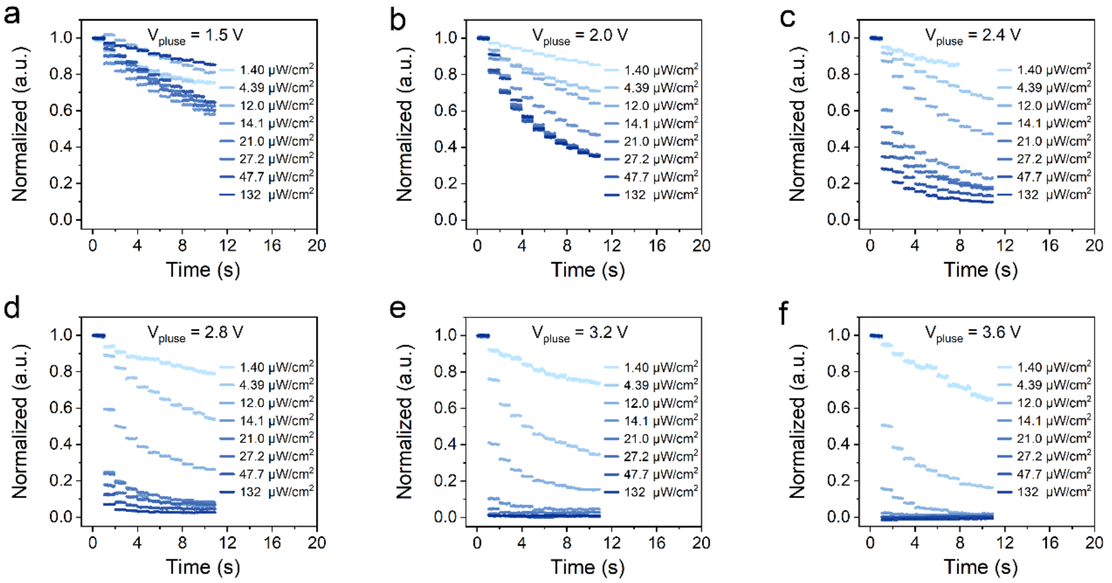

Fig. S24 Effect of voltage pulses of different amplitudes on the photoconductivity of $In_2S_{3-X}As_X$.

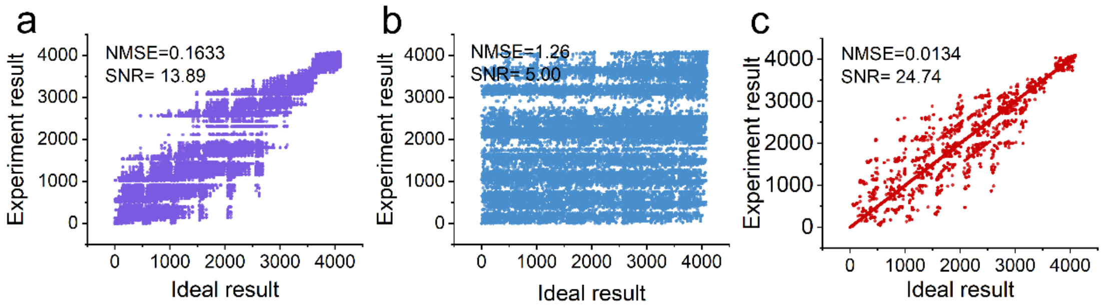

Fig. S25 Comparison of coding accuracy using different data. (a) The accuracy of information encoding using the sum of the output currents I_{read1} under the original photoconductance. (b) The accuracy of information encoding using the sum of the output currents I_{read2} after the electrical pulse programming. (c) The accuracy of encoding 12–bits binary information using I_{read1} and I_{read2}.

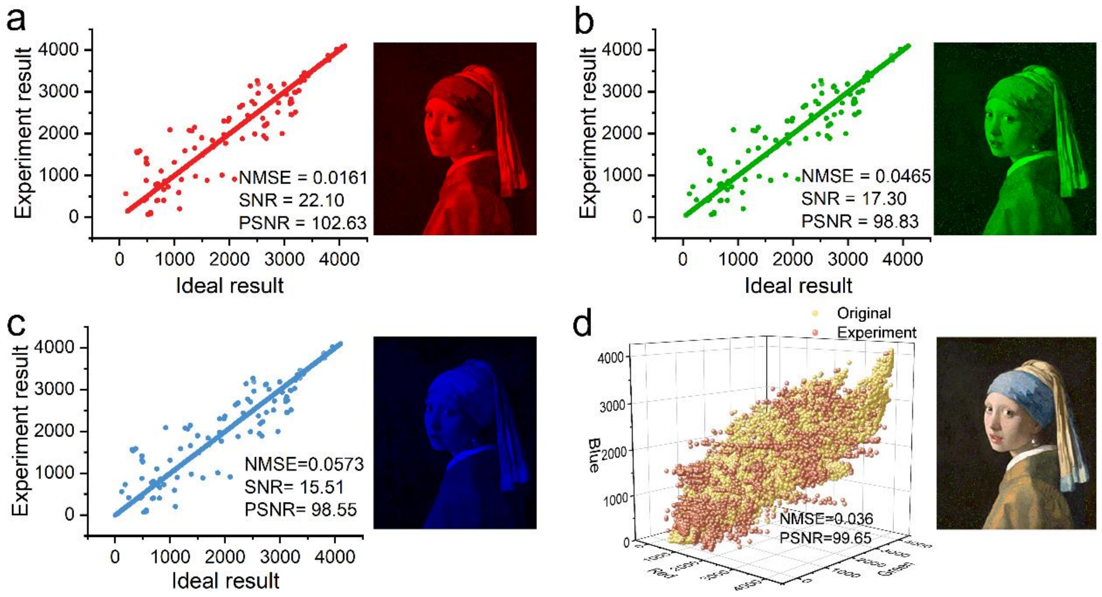

Fig. S26 Restored Girl with A Pearl Earring image based on ROMA. The coding accuracy of the R (a), G (b), and B (c) color spaces of the Girl With A Pearl Earring. (d) The photo of Girl with A Pearl Earring recovered based on ROMA encoding.

Table S1. Comparison of the different memristor devices.

Materials	Wavelength Range (nm)	Pulse width	Light intensity (mW/cm^2)	Energy consumption/spike (Light & Electric)	Ref
$Pt/Ga_2O_3/Ag$	254-365	3s	1.8	0.42 μJ	17
$TiN/CeO_2/$ ZnO/ITO	405-808	2s	20	187 pJ	18
Cr_2O_3/VO_2	-	1 μs	-	3.9 nJ	19
TiOx/ZnO	405-650	10 ms	0.03	2nJ	20
$Pt/Pb(Zr_{0.2}Ti_{0.8})O_3/SrRuO_3$	365	10 μs	14	56 nJ	21
$In_2S_{3-X}As_X$	450-635	100 ms	0.038	3.30 pJ	This Work

Table S2. A Comparative Analysis of Persistent Photoconductivity (PPC) Models for $In_2S_{3-X}As_X$

Model	LLR [9]	MB [8]	RLPF [10]
**Mechanistic Features**	(1) Deep-level traps (DX centers) capture carriers with significant lattice relaxation;<br>(2) Forms an energy barrier requiring thermal activation for recombination.	(1) Carriers separated/trapped at macroscopic interfaces (grain boundaries, heterojunctions);<br>(2) Surface adsorption or barrier-driven.	Random spatial potential fluctuations trap carriers in disordered media, leading to PPC.
**Applicable Conditions**	(1) Single□crystal or highly crystalline semiconductors<br>(2) Presence of dopants/defects that form DX centers	(1) Polycrystalline or amorphous materials with abundant interfaces<br>(2) Sensitive to surface environment	(1) Amorphous or heavily defective materials;<br>(2) Non-uniform defect/dopant distribution.
**Performance in $In_2S_{3-X}As_X$ System**	(1) HRTEM & XRD confirm single-crystal nanowires (Fig. S1d, S20b);<br>(2) As doping + oxygen vacancies yield DX centers;<br>(3) 404 meV capture barrier & temperature-dependent PPC recovery match LLR predictions (Fig. 3i, 3h).	PPC persists in vacuum (Fig. S7a), but no grain boundaries or heterojunctions in single-crystal nanowires.	(1) EDS mapping shows uniform doping (Fig. S1b);<br>(2) Raman symmetry confirms high crystallinity (Fig. 3a).
**Limitations / Inapplicability**	None for this system; fully consistent with data.	(1) Absence of grain boundaries or heterojunctions<br>(2) Surface effects ruled out.	High crystallinity and uniform doping negate random fluctuations.

REFERENCES

1. Delley B. An all–electron numerical method for solving the local density functional for polyatomic molecules. *J Chem Phys* 1990; **92**: 508–517.
2. Perdew JP, Burke K, Ernzerhof M. Generalized gradient approximation made simple. *Phys Rev Lett* **996**; 77: 3865–3868.
3. Datta A, Gorai S, Chaudhuri SS. Synthesis and characterization of sol–gel derived Mn^{2+}–doped In_2S_3 nanocrystallites embedded in a silica matrix. *J Nanopart Res* 2006; **8**: 919–926.
4. Lucena R, Aguilera I, Palacios P *et al.* Synthesis and spectral properties of nanocrystalline V–substituted In_2S_3, a novel material for more efficient use of solar radiation. *Chem Mater* 2008; **20**: 5125–5127.
5. Ho CH. Enhanced photoelectric–conversion yield in niobium–incorporated In_2S_3 with intermediate band. *J Mater Chem C* 2011; **21**: 10518–10524.
6. Mathew M, Rao SMS, Prasad V *et al.* Anomalous behavior of silver–doped indium sulfide thin films. *J Appl Phys* 2006; **100**: 033504.
7. Sumanth A, Ganapathi KL, Rao MS *et al.* A review on realizing the modern optoelectronic applications through persistent photoconductivity. *J Phys D: Appl Phys* 2022; **55**: 393001.
8. Choi HS, Jeon S. Field–induced macroscopic barrier model for persistent photoconductivity in nanocrystalline oxide thin–film transistors. *Appl Phys Lett* 2014; **104**: 133507.
9. Lang DV, Logan RA. Large–lattice–relaxation model for persistent photoconductivity in compound semiconductors. *Phys Rev Lett* 977; **39**:

635–639.

10. Abelenda A, Calvo F, Izquierdo R *et al.* Anomalous persistent photoconductivity in Cu_2ZnSnS_4 thin films and solar cells. *Sol Energy Mater Sol Cells* 2015; **137**: 164–168.

11. Singh S, Mohapatra YN. Persistent photocurrent (PPC) in solution–processed organic thin–film transistors: mechanisms of gate voltage control. *J Appl Phys* 2016; **120**: 045501.

12. He W, He J, Liu C *et al.* A multi□input light□stimulated synaptic transistor for complex neuromorphic computing. *J Mater Chem C* 2019; **7**: 12523–12531.

13. Lin JY, Dissanayake A, Jiang HX. Electric–field–enhanced persistent photoconductivity in a $Zn_{0.02}Cd_{0.98}$ Te semiconductor alloy. *Phy Rev B* 1992; **46**: 3810–3816.

14. Gurwitz R, Cohen R, Shalish I. Interaction of light with the ZnO surface: photon–induced oxygen "breathing," oxygen vacancies, persistent photoconductivity, and persistent photovoltage. *J Appl Phys* 2014; **115**: 033701.

15. Jeon S, Son J, Lee H *et al.* Gated three–terminal device architecture to eliminate persistent photoconductivity in oxide semiconductor photosensor arrays. *Nat Mater* 2012; **11**: 301–305.

16. Lu J, Luo L, Xu L *et al.* Epitaxial growth of large–scale In_2S_3 nanoflakes and the construction of a high–performance In_2S_3/Si photodetector. *J Mater Chem C* 2019; **7**: 12104–12113.

17. Tan H, Chen Y, Liu Y *et al.* Versatile optoelectronic memristor based on wide–bandgap Ga_2O_3 for artificial synapses and neuromorphic computing. *Adv Mater* 2022; **34**: 2109923.

18. Zhao Q, Wang Y, Li Y *et al.* Low energy consumption photoelectric memristors with multi–level linear conductance modulation in artificial visual systems application. *Nano-Micro Lett* 2022; **14**: 191.

19. Wu C, Kim TW, Park J *et al.* Crossmodal sensory neurons based on high–performance flexible memristors for a human–machine in-sensor computing system. *Nat Electron* 2022; **5**: 654–64.

20. Zhou Y, Zhang J, Chen Z *et al.* Fully integrated multi–mode optoelectronic memristor array for diversified in–sensor computing. *Sci Adv* 2023; **9**: eadf8491.

21. Cui B, Li K, Feng W *et al.* Ferroelectric photosensor network: an advanced hardware solution to real–time machine vision. *Nat Commun* 2022; **13**: 1707.
